# Discriminating Post-Transplant Rejection from Infection by Detecting TCR-CD3 Oligomerization on Extracellular Vesicles Using a Ratiometric Caliper Probe

**DOI:** 10.1021/jacs.6c07971

**Published:** 2026-05-28

**Authors:** Wen Yin, Haitian Chen, Shu Xiao, Jun Zheng, Xuegang Zhao, Linda Fan, Xing Lv, Haijin Lv, Qing Yang, Jia Yao, Xiaofeng Yuan, Yang Yang, Mo Yang

**Affiliations:** † Department of Biomedical Engineering, 26680 The Hong Kong Polytechnic University, Hunghom, Hong Kong 999077, China; ‡ Department of Hepatic Surgery and Liver Transplantation Centre, 144991 The Third Affiliated Hospital of Sun Yat-sen University, Guangzhou 510630, China; § Guangdong Provincial Key Laboratory of Liver Disease Research, The Third Affiliated Hospital of Sun Yat-sen University, Guangzhou 510630, China; ∥ Department of General Intensive Care Unit, The Third Affiliated Hospital of Sun Yat-sen University, Guangzhou 510630, China; ⊥ Surgical and Transplant ICU, Department of Anesthesiology and Critical Care, The Third Affiliated Hospital of Sun Yat-sen University, Guangzhou 510630, China; # The Hong Kong Polytechnic University Shenzhen Research Institute, Shenzhen 518000, China; ¶ Joint Research Center of Biosensing and Precision Theranostics, The Hong Kong Polytechnic University, Kowloon, Hong Kong 999077, China

## Abstract

Post-transplant rejection and infection remain significant obstacles to long-term patient survival. However, there is currently no standardized assay to simultaneously assess an individual patient’s risk for both complications. Here, we demonstrate that T cell receptor (TCR)-CD3 oligomers on extracellular vesicles (EVs) represent a promising biomarker for acute cellular rejection. To leverage this, we developed a caliper-shaped aptamer probe to quantify the ratio of TCR-CD3 oligomeric to monomeric EVs derived from CD8+ cytotoxic T cells. This method inherently normalizes the differences in overall EV abundance and minimizes variability arising from plasma input volume (5–15 μL) and storage conditions, thereby ensuring highly robust results. In murine models, the assay yielded markedly higher fluorescence ratios in mice experiencing allograft rejection than in those with infection (mean 0.26 vs 0.14). Crucially, in a clinical cohort of 34 transplant recipients, the assay reliably distinguished rejection and infection with overlapping clinical manifestation and also accurately identified rejection even in patients with concurrent rejection and infection. At a diagnostic cutoff value of 0.69, the assay demonstrated robust performance, with an area under the curve of 0.85, corresponding to a sensitivity of 71% and a specificity of 90%. Ultimately, this approach offers a minimally invasive tool for routine immune monitoring, with the potential to guide immunosuppressive therapy and reduce the reliance on biopsies.

## Graphical abstract

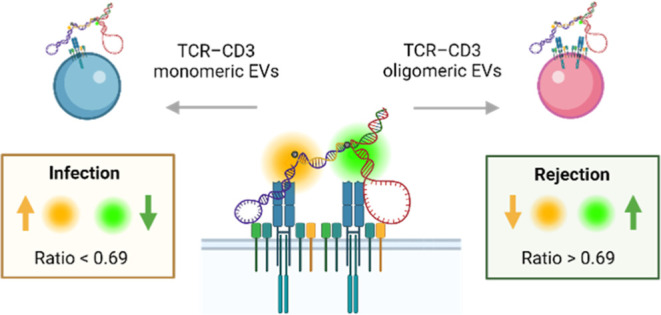

## Introduction

Organ transplantation remains the only definitive treatment for end-stage organ failure. However, rejection and infection are major postoperative complications to early graft dysfunction, transplant failure, and recipient mortality.^1^ Immunosuppressive agents are essential for preventing allograft rejection, yet their use inevitably increases the risk of infection. Successful organ transplantation necessitates meticulous attention to the balance between graft rejection and infection.^2^ A key challenge lies in the lack of standardization and validation of commercial assays to evaluate an individual’s immune status in relation to these risks. Although tissue biopsy and microbiological assays are the gold standards for diagnosing rejection and infection, respectively, their clinical utility is heavily compromised by their invasiveness, risk of patient harm, and time-consuming procedures.^3,4^ Conventional markers of allograft dysfunction and inflammatory markers are not sufficiently sensitive nor specific for timely differentiate rejection from infection, with their perturbations often trailing behind immunological injury.^5–7^ In practice, clinicians must still rely on experience and judgment to guide management decisions.

The distinct mechanisms underlying rejection and infection have been well characterized, reflecting the dominance of adaptive and innate immunity, respectively. During acute cellular rejection, donor-derived antigenic peptides presented by major histocompatibility complex (pMHC) initiate allorecognition by engaging the T cell receptor (TCR)-CD3 complex on recipient T cells, leading to the activation of CD8+ cytotoxic T cells that directly kill antigen-expressing cells via cytotoxic granules.^8^ In contrast, infections are often caused by the reactivation of latent pathogens originating from either the recipient or the donor. The immune response to these infections is predominantly driven by innate immune mechanisms, such as macrophage phagocytosis, dendritic cell (DC) activation, and neutrophil infiltration.^9^ Thus, there is a strong theoretical rationale for using T cell activation status as a potential biomarker to distinguish rejection from infection in transplant recipients. To date, only the Cylex Immuknow assay has been approved by Food and Drug Administration (FDA) to evaluate infection and rejection risk by measuring T cell function,^10^ however, several studies have reported its limited accuracy in differentiating between these two conditions.^11,12^ Therefore, developing a more precise and specific strategy to monitor T cell activation after transplantation remains a challenge.

Previous studies have elucidated that full activation of T cells requires both aggregation and conformational changes of the TCR-CD3 complexes.^13,14^ Specifically, the proximity of neighboring TCR-CD3 complexes at the T cell–antigen-presenting cell (APC) interface markedly enhances signal transduction. The nanoscale organization of multimeric pMHC has been reported to increase TCR binding avidity, prolong interaction dwell time, and promote TCR clustering, with dense nanoclusters exhibiting the highest triggering efficiency.^15–17^ This spatial organization is established within the specialized junction between T cells and APCs, known as the immunological synapse, where centrally accumulated TCR-CD3 complexes are enriched on extracellular vesicles (EVs) that bud from the synaptic center.^18^ Notably, activated T cells release increased numbers of EVs bearing TCR-CD3 complexes compared to their unactivated counterparts.^19,20^ Building on these observation, our previous work identified two distinct T cell-derived EV subpopulations in the plasma of mice undergoing acute rejection, characterized by the presence of either monomeric or oligomeric TCR-CD3 complexes.^21^ Based on these findings, we propose that EV signatures defined by TCR-CD3 oligomerization may serve as a more precise indicator of T cell activation in transplant recipients, with the potential to distinguish rejection from infection.

In this study, we develop a ratiometric caliper-shaped probe to assess T cell activation by quantifying TCR-CD3 oligomerization on the surface of circulating EVs (Scheme 1). The probe is structurally engineered with two CD3 aptamer switches featuring distinct apparent affinities, precise spatial arrangement, and a tailored interaptamer distance to selectively discriminate monomeric from oligomeric TCR-CD3 complexes. Target engagement generates differential yellow and green fluorescence signals, enabling ratiometric evaluation of TCR-CD3 oligomerization. In vitro allogeneic T cell-APC coculture assays revealed an increased release of EVs bearing oligomeric TCR-CD3 from activated CD8+ T cells. In mouse models, the probe detected elevated oligomer-to-monomer fluorescence ratios during allograft rejection and reduced ratios during septic conditions. Clinically, this approach distinguished transplant patients with rejection from those with infection by directly analyzing 5 μL of plasma within 2 h using a microplate reader, offering a rapid, portable, and scalable workflow. Overall, this work establishes a caliper probe-based strategy for profiling TCR-CD3 oligomerization on plasma EVs, highlighting its potential as a minimally invasive biomarker for immune monitoring and precision management of immunosuppressive therapy following transplantation.

**Scheme 1:**
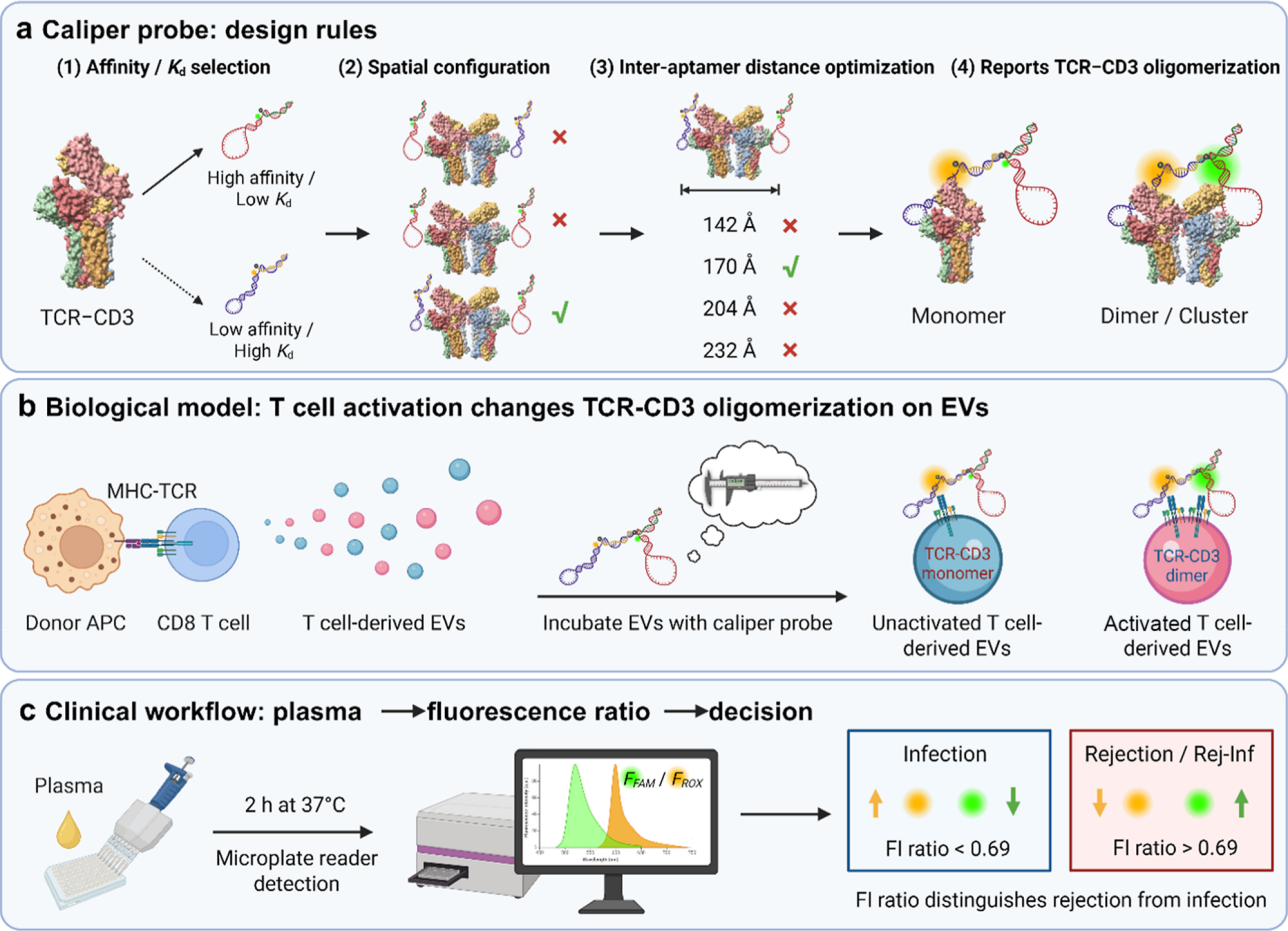
Caliper Probe Detects TCR-CD3 Oligomerization on EVs to Distinguish Rejection from Infection by Profiling Ratiometric Fluorescence Signals^a^–^c^

a The TCR-CD3 affinity, spatial configuration, and inter-aptamer distance optimization of the two aptamer switch probes were optimized to selectively bind TCR-CD3 monomer and dimer and trigger the emission of ROX (yellow) and FAM (green) fluorescence, respectively.
b Upon MHC recognition to donor APC in the cell and mouse model, CD8+ T cells activate and secret EVs bearing dimeric TCR-CD3, which can be evaluated by the caliper probe.
c The ratiometric fluorescence signals of plasma can be rapidly quantified using a microplate reader for high-throughput analysis. The FI ratio is relatively low (< 0.69) in infection patients and high (> 0.69) in rejection or patients coexisting rejection and infection (Rej-Inf).

## Results

### Structural Optimization of Caliper Probes Enables Differential Responses to TCR-CD3 Oligomerization on EVs

To establish a model of T cell-derived EVs, EVs secreted by Jurkat cells were isolated via ultracentrifugation. Transmission electron microscopy (TEM) characterization revealed spherical nanoscale vesicles with well-defined lipid bilayer structures (Figure 1a). Western blot analysis confirmed the presence of EV protein markers, CD63 and CD9,^22^ along with the T cell-specific surface protein CD3 (Figure 1b). Furthermore, nanoflow cytometry (nFCM) indicated a mean particle diameter of 76.2 ± 22.0 nm (Figure 1c) and a CD3-positive ratio of 19.7% (Figure 1d), validating these vesicles as suitable CD3 + EVs for subsequent assay development.

**Figure 1:**
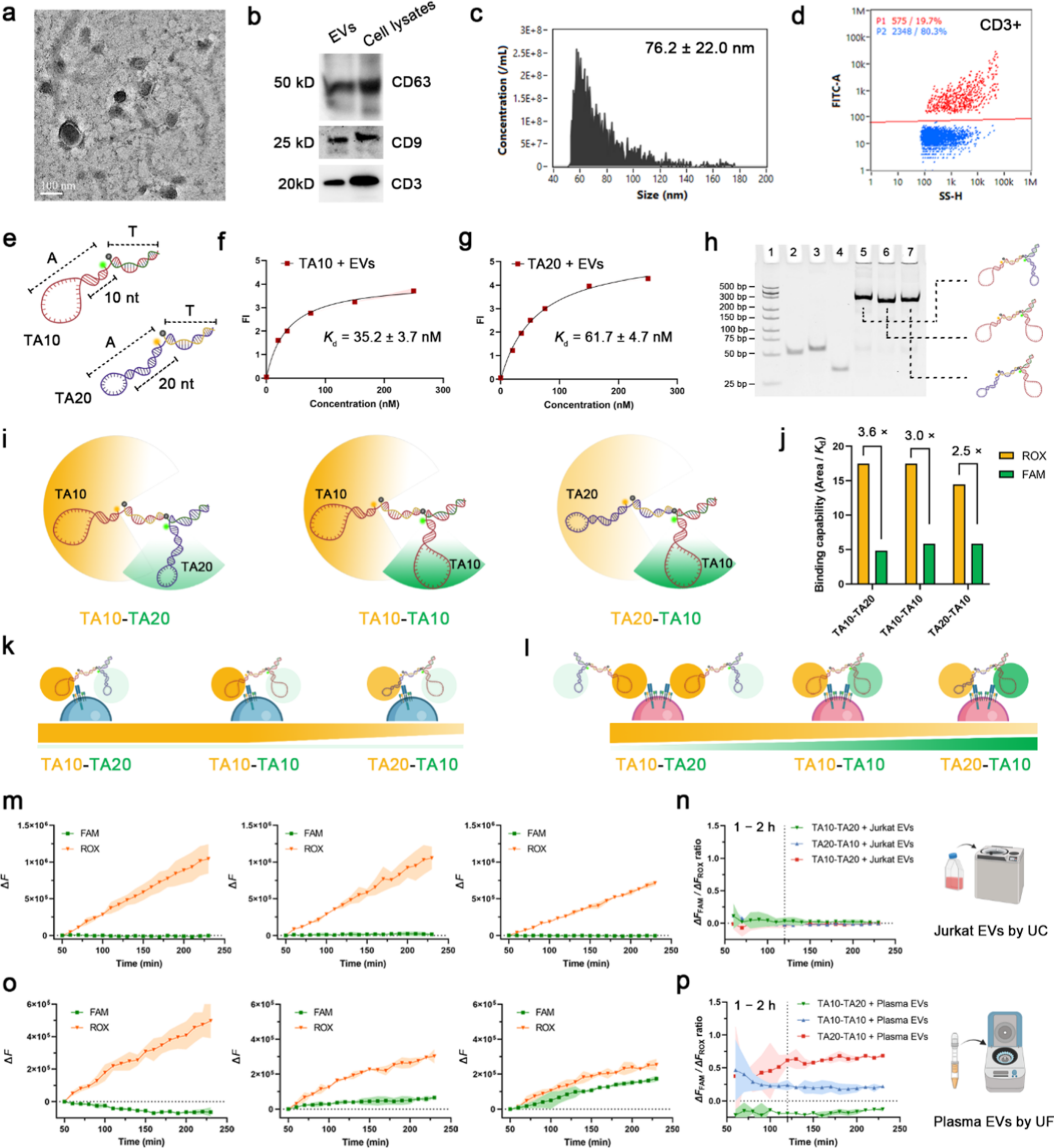
Structural design and functional characterization of caliper probes for differential detection of TCR-CD3 oligomerization on EVs. (a) TEM image of Jurkat EVs. (b) Detection of CD63, CD9, and CD3 expressed on Jurkat EVs and cells by Western blot assay. (c) Size distribution of Jurkat EVs measured by nFCM. (d) The CD3 positive proportion of Jurkat EVs measured by nFCM. (e) Structure diagrams of TA10 and TA20. Determination of the *K*_d_ for TA10 (f) and TA20 (g) via fluorescence saturation curves. (h) Validation of the hybridization of caliper probes via domain length by PAGE assay. Lane 1: DNA markers; lane 2: A10-T1*; lane 3: A20-T2*, lane 4: T1-T2, lane 5: TA10-TA20, lane 6: TA10-TA10, lane 7: TA20-TA10. (i) Structure diagrams of TA10-TA20, TA10-TA10, and TA20-TA10. The circular sectors illustrate the binding accessibility of the terminal and central probes, respectively. (j) Binding capability of probes at different positions, as quantified by the ratio of binding area to *K*_d_. Schematic representation of the binding interactions between caliper probes and TCR-CD3 monomeric (k) or oligomeric (l) EVs, along with fluorescence signal changes. (m) Fluorescence kinetic curves of caliper probes interacting with Jurkat EVs isolated by ultracentrifugation (UC) (mean ± s.d., *n* = 3). (n) The Δ*F*_FAM_/Δ*F*_ROX_ ratio reflects the relative fluorescence response of the caliper probes upon binding to Jurkat EVs. (o) Fluorescence kinetic curves of caliper probes interacting with plasma EVs isolated by ultrafiltration (UF) (mean ± s.d., *n* = 3). (p) The Δ*F*_FAM_/Δ*F*_ROX_ ratio reflects the relative fluorescence response upon interaction with plasma EVs.

As illustrated in Figure 1e, the aptamer switch probe consists of three elements: a CD3-binding aptamer (A), a DNA strand partially complementary to the aptamer, and a tethering domain (T) for anchoring. A fluorophore and quencher are covalently conjugated to the termini of A and T, respectively, keeping the probe in a quenched (“off”) state in the absence of target. Upon binding to CD3, the aptamer undergoes conformational rearrangement that disrupts intramolecular hybridization, spatially separating the quencher from the fluorophore restoring fluorescence (“on” state).^23^ The efficiency of fluorescence recovery depends critically on the length of the complementary sequence, which governs aptamer dissociation kinetics.^24^ Extending the complementary sequence from 10 nt (TA10) to 20 nt (TA20) resulted in an elevated dissociation constant (*K*_d_) for CD3 + EVs (from 35.2 ± 3.7 nM to 61.7 ± 4.7 nM), corresponding to a gradual reduction in binding affinity (Figures 1f,g and S1). To evaluate the detection sensitivity of the aptamer switch probes, we quantified the fluorescence responses of TA10 and TA20 to increasing concentrations of CD3 + EVs. The results demonstrated excellent linearity (*R* ^2^ > 0.995) in the range of 1.6 × 10^8^ to 8.0 × 10^9^ particles mL^–1^ (Figure S2). The limit of detection (LOD) for both probes was determined to be 2.2 × 10^8^ particles mL^–1^. Based on their favorable detection capabilities and tunable affinities, TA10 (high affinity) and TA20 (low affinity) were selected for further investigations.

Caliper probes were constructed by pairing TA10 and TA20 in three spatial configurations, and their successfully assembly was validated via polyacrylamide gel electrophoresis (PAGE) analysis (Figure 1h). The terminal aptamer possesses greater conformational freedom than the constrained central aptamer, with their binding ranges modeled as 270° and 90° sectors, respectively (Figure 1i). The binding capability was defined as the ratio of the accessible sector area to the *K*_d_ values, serving as a composite metric of spatial and affinity contributions. As calculated in Figure 1j and Table S1, the terminal probe exhibited 3.6-, 3.0-, and 2.5-fold higher binding capabilities than the central probe in the TA10-TA20, TA10-TA10, and TA20-TA10 configurations, respectively. Based on this design, we hypothesized that monomeric TCR-CD3 would preferentially engage the terminal probe, activating ROX fluorescence while leaving the central probe largely unresponsive (Figure 1k). For oligomeric TCR-CD3 (Figure 1l), the binding behaviors vary by configuration. In the TA10-TA20 configuration, the terminal probe’s 3.6-fold higher binding capability drives the interaction, primarily activating ROX fluorescence from both ends of the dimer. In TA10-TA10, the central probe’s enhanced binding capability increases its likelihood of engaging a second dimer subunit, thereby triggering FAM fluorescence. Finally, in TA20-TA10, the reduced binding disparity promotes a more balanced interaction, potentially enabling simultaneous activation of both ROX and FAM fluorescence upon dimer binding.

To evaluate fluorescence kinetics, Jurkat EVs and mouse plasma EVs were employed as models for monomeric and oligomeric TCR-CD3, respectively. To eliminate interference from intermolecular hybridization, the net signal change was calculated as Δ*F* = *F* – *F*_blank_, using the EV-free fluorescence at 50 min as the background (Figure S3). For Jurkat EVs, ROX fluorescence increased over time, with TA10-TA20 and TA10-TA10 generating stronger signals than TA20-TA10, while FAM signals remained negligible (Figure 1m). Consequently, the Δ*F*_FAM_/Δ*F*_ROX_ ratios approached zero for all probes (Figure 1n), confirming exclusive activation of the terminal aptamer by monomeric targets. Conversely, plasma EVs isolated via ultrafiltration (mean diameter of 169 ± 51 nm, Figure S4) induced distinct responses. TA10-TA20 showed selective ROX activation, whereas TA10-TA10 exhibited increased FAM and reduced ROX signals. Notably, TA20-TA10 generated near-equal ROX and FAM signals, indicating successful dual-site recognition (Figure 1o). Reaching equilibrium after 2 h, TA20-TA10 achieved the highest ratio (0.63) compared to TA10-TA10 (0.21) and TA10-TA20 (−0.18) (Figure 1p), establishing it as the optimal configuration. Practically, while designed for dimers, this dual-fluorescence signal reflects the general oligomerization or microclustering of TCR-CD3.

To verify the specificity and reliability of the caliper probes, CD3-negative EVs and EV-depleted plasma were employed as controls. Both yielded negligible fluorescence compared to CD3-positive EVs (Figure S5), confirming the good specificity of the probes and resistance to interference from free plasma proteins. Building on this, the assay was conducted directly in plasma. The resulting fluorescence kinetics and Δ*F*_FAM_/Δ*F*_ROX_ ratios for all three caliper probes closely mirrored those obtained from isolated EVs (Figure S6), indicating that the probes retain functional integrity in complex biofluids. This compatibility with unprocessed samples supports the feasibility of direct, minimal-preparation detection of TCR-CD3 oligomerization in clinical settings.

### Caliper Probes Identify TCR-CD3 Oligomeric EVs as Indicators of T Cell Activation

To mimic the physiological stimulation of T cells by alloantigen-presenting cells, a mixed lymphocyte reaction (MLR) model was established using allogeneic CD8+ T cells and dendritic cells (DCs) (Figure 2a). Briefly, bone marrow-derived DCs were generated from Balb/c mice and differentiated for 8 days to achieve approximately 90% purity. After maturation, flow cytometry confirmed significant upregulation of MHC II, CD80, and CD86, validating their antigen-presenting capacity (Figure S7). CD8+ T cells were isolated from the spleen of C57BL/6 mice using magnetic beads (91.6% purity, Figure S8). Upon coculturing these allogeneic T cells with DCs, T cell proliferation and effector functions were evaluated in vitro. Flow cytometry revealed a marked upregulation of CD25 on the T cells (Figure 2b), while carboxyfluorescein succinimidyl ester (CFSE) staining confirmed strong T cell proliferation (Figure 2c). Furthermore, increased secretion of interferon-gamma (IFN-γ) was observed in the coculture groups (Figure 2d), collectively indicating robust T cell activation in this MLR model.

**Figure 2:**
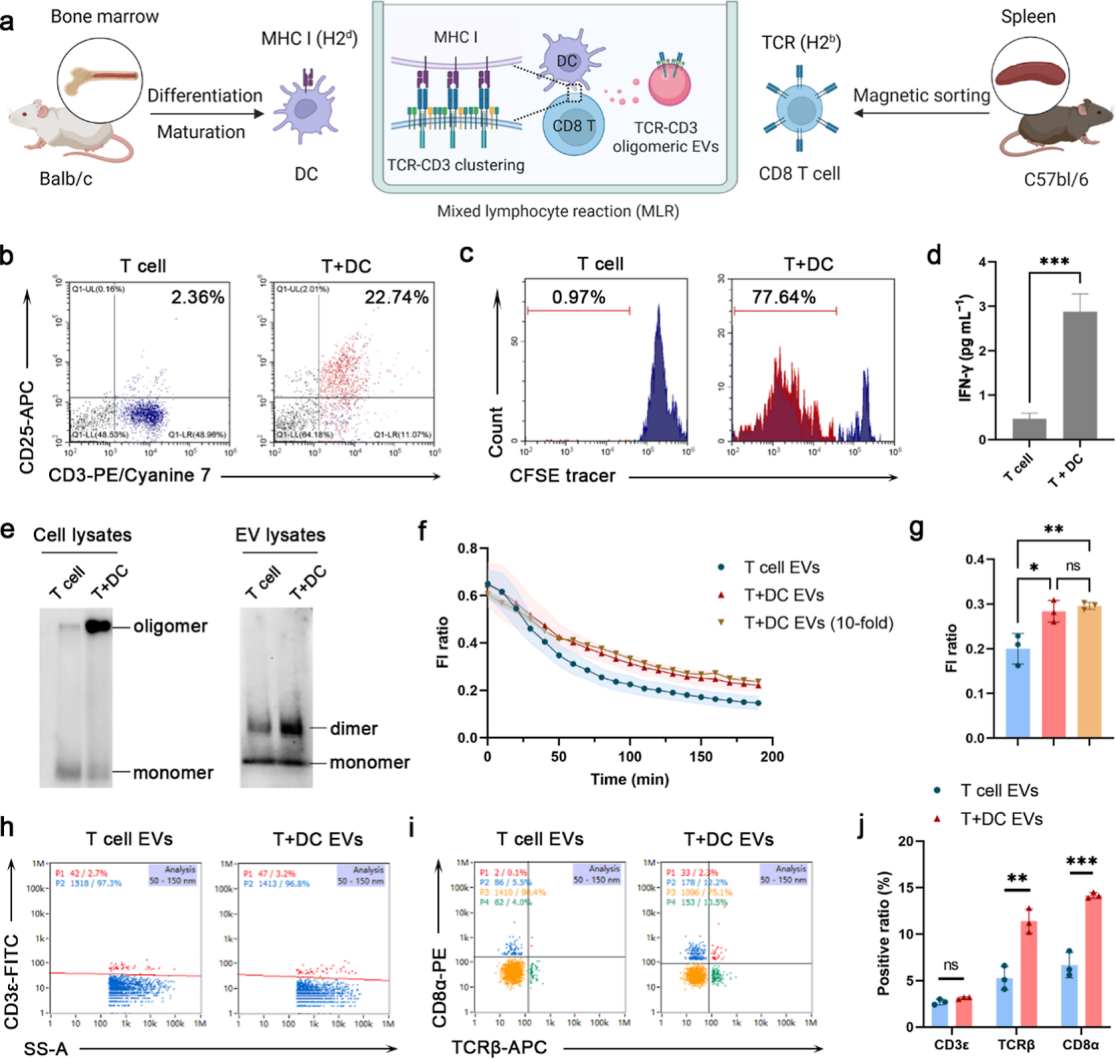
Detection performance of the caliper probe for TCR-CD3 monomeric and oligomeric EVs in the in vitro allogeneic T cell activation model. (a) Schematic illustration of the MLR model between allogeneic T cells and DCs. (b) Flow cytometric analysis of CD3 and CD25 expression on T cells alone or coculture with DCs. (c) Assessment of T cell proliferation via CFSE staining alone or coculture with DCs. (d) Quantification of IFN-γ secretion in the cell culture supernatants by ELISA alone or coculture with DCs (*n* = 3). Statistical significance was determined using an unpaired two-tailed *t* test. (e) Evaluation of TCR-CD3 protein oligomerization in cell and EV lysates from the T cell and T + DC coculture groups by BN-PAGE and anti-CD3ε immunoblotting. Real-time FI ratio curves (f) and the corresponding 2 h FI ratio (g) of the EVs in T cell, T + DC, and 10-fold T + DC groups, as detected by the caliper probe (*n* = 3). Statistical significance was analyzed using one-way ANOVA followed by Tukey’s multiple comparisons test. The *n*FCM analysis of the abundance of CD3ε (h), CD8α, and TCRβ (i) of T cell- and T + DC-derived EVs. (j) The positive ratios of CD3ε, CD8α, and TCRβ of T cell- and T + DC-derived EVs (*n* = 3). Statistical significance was determined using multiple unpaired two-tailed *t* tests. ns = no significance; *p < 0.05; **p < 0.01; ***p < 0.001. Data is presented as mean ± s.d.

To analyze the size distribution of TCR-CD3 complexes on T cells and EVs, Blue Native polyacrylamide gel electrophoresis (BN-PAGE) was employed using the mild detergent Brij96, which extracts and preserves intact TCR-CD3 oligomers.^25^ As shown in Figure 2e, in the cell lysates, the resting T cell group predominantly exhibited a monomeric TCR-CD3 band with only a faint oligomeric band. Upon allogeneic stimulation, the T + DC group showed a dramatically enhanced oligomeric TCR-CD3 band accompanied by a weakened monomeric band. As expected, the DC control group showed no detectable TCR-CD3 bands (Figure S9). Crucially, this pattern was mirrored in the EV lysates: while EVs from both the T and T + DC groups contained monomeric TCR-CD3, the EVs derived from the T + DC group exhibited a significantly stronger dimeric TCR-CD3 band than those from resting T cell, driven by the allogeneic activation. Together, these findings provide direct, orthogonal biophysical evidence that T cells stimulated by allogeneic DCs actively secret EVs enriched with dimeric TCR-CD3.

The caliper probe was then applied to detect the TCR-CD3 oligomerization on EVs, revealing that the FI ratio (calculated as *F*_FAM_/*F*_ROX_) of T + DC EVs was 0.28 ± 0.02, which was significantly higher than that of the resting T cell group (0.20 ± 0.04) (Figure 2f,g). Moreover, when the concentration of T + DC EVs was increased 10-fold to elevate CD3 abundance, the caliper probe reported proportional increase in both ROX and FAM fluorescence signals (Figure S10), while the FI ratio remained essentially unchanged (0.29 ± 0.01). As characterized by *n*FCM, EVs secreted by resting T cells and the T + DC coculture exhibited similar sizes of approximately 60 nm (Figure S11) and a comparable CD3-positive fraction of about 3% (Figure 2h). These results suggest that the FI ratio measured by the caliper probe predominantly reflects the oligomerization state of TCR-CD3, rather than variations in overall CD3 abundance.

Furthermore, the expression levels of TCRβ and CD8α on EVs were analyzed. Compared to the background nonspecific antibody binding on DC-derived EVs (Figure S12), the positivity rates for TCRβ and CD8α on T + DC EVs (11.4% and 14.2%, respectively) were approximately double those on EVs from resting T cells (5.3% and 6.7%). The discrepancy between TCR and CD3 detection rates on EVs is likely due to steric hindrance caused by the large size of IgG antibodies. Notably, the double-positive rate (TCRβ+ CD8α+) on T + DC EVs (2.3%) was nearly 20-fold higher than that on T cell EVs (0.1%) (Figure 2i,j). Biologically, T cells exert their effector functions through the formation of an immunological synapse, a specialized junction characterized by a highly organized spatial pattern known as the supramolecular activation cluster (SMAC).^26^ Upon antigen recognition, the TCR and the coreceptor CD8 undergo coordinated coclustering and polarize toward the central SMAC (cSMAC).^27^ Recent evidence indicates that intercellular communication at this interface is extensively mediated by the generation and release of distinct EVs from the effector T cells.^28^ During this process, CD8+ cytotoxic T cells secrete EVs containing perforin and granzyme B into the synaptic cleft to eliminate target cells.^29^ Concurrently, they release EVs enriched with TCRs derived from the cSMAC, which can contribute to signal propagation or attenuation.^19,20,28,30^ Therefore, the concurrent enrichment of these components on EVs in the coculture group strongly suggests that these vesicles originate from the immunological synapse of activated CD8+ T cells, rather than from nonspecific membrane shedding. This activation-driven release mechanism provides a mechanistic basis for the use of TCR-CD3 oligomeric EVs as biomarkers of allogeneic T cell activation.

### Distance-Dependent Detection of Rejection Using Caliper Probes in Mouse Models

To induce an in vivo rejection response, an allogeneic skin transplantation mouse model was established by grafting full-thickness dorsal skin from Balb/c donor mice onto C57BL/6 recipients, while autologous skin graft was applied as a nonrejection control group.^31^ The transplanted skin in the autograft group showed mild erythema and gradually heal, whereas the allograft group displayed severe clinical signs including erythema, edema, ulceration, subsequent hardening, and scab formation (Figure 3a). By day 15, over 80% of the graft tissue was necrotic, indicating complete rejection. Hematoxylin–eosin (H & E) staining alongside immunohistochemical (IHC) staining for CD3 (stained brown and marked by red arrows), revealed extensive inflammatory cells and T cell infiltration at the host–donor interface in the allograft group (Figures S13 and 3b). This suggests that the primary rejection mechanism in allogeneic transplantation is T cell-mediated rejection. Additionally, flow cytometric analysis of peripheral blood revealed a progressive decline in the CD4+/CD8+ T cell ratio, dropping from 2.7 in naive mice to 2.0 in autograft recipients, and further to 1.4 in the allograft group (Figures S14 and 3c). This highlights the dominant role of CD8+ T cells in allogeneic transplantation and is consistent with previous reports associating a reduced CD4+/CD8+ ratio to increased risk of rejection.^32^

**Figure 3:**
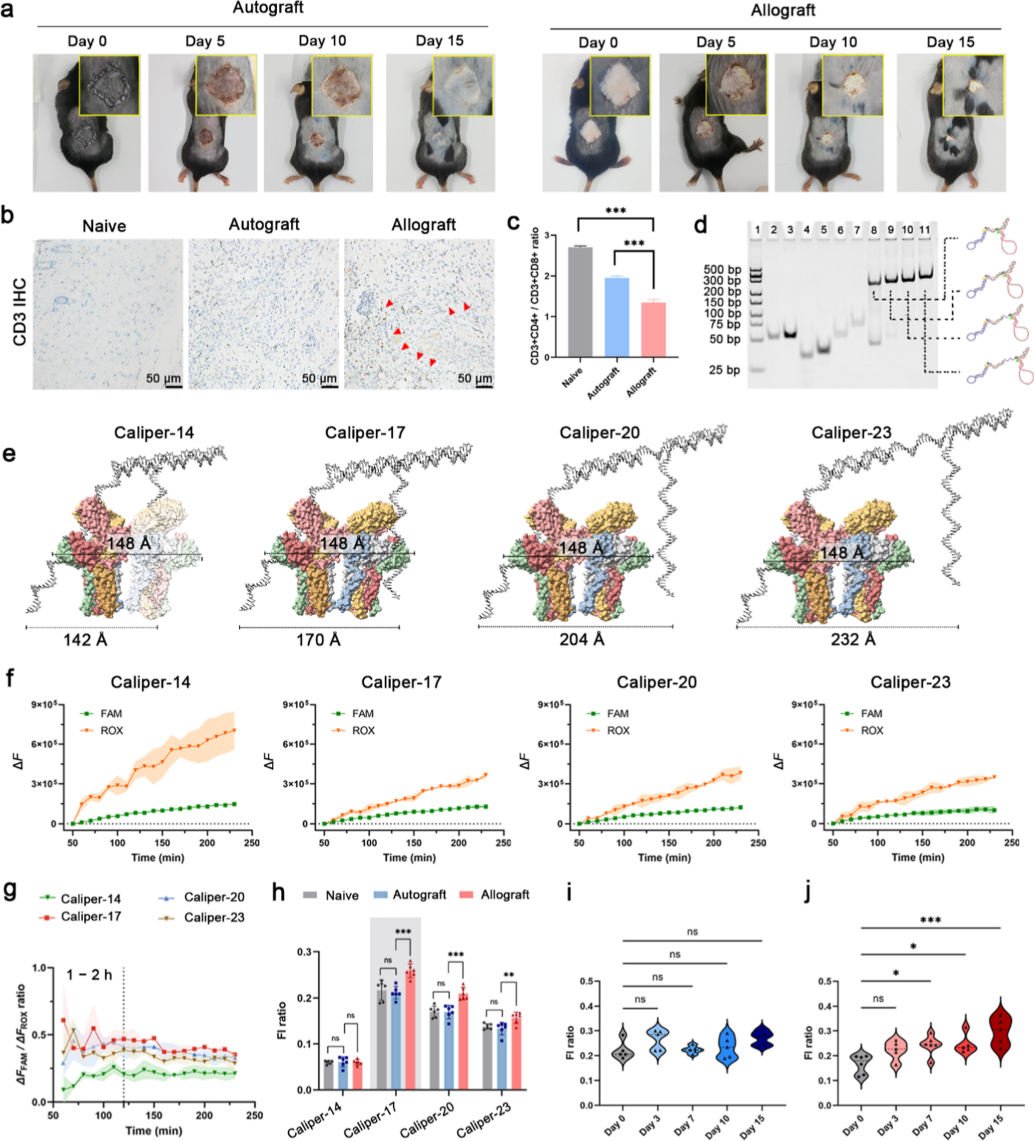
Detection of T cell-mediated rejection in a skin transplant mouse model using caliper probes with varying interprobe distances. (a) Gross morphological changes of the skin grafts in the autograft and allograft groups. Insets: enlarged images of the skin grafts. (b) Representative CD3 IHC staining images of the skin tissues from naive, autograft, and allograft mice. (c) Flow cytometric analysis of CD4+/CD8+ T cell ratio in the peripheral blood of naive, autograft, and allograft mice (*n* = 3). Statistical significance was determined using a one-way ANOVA followed by Tukey’s multiple comparisons test. (d) Validation of the hybridization of caliper probes with different interprobe spacing using a PAGE assay. Lane 1: DNA markers; lane 2: A10-T1*; lane 3: A20-T2*, lane 4: T1-T3, lane 5: T1-T2, lane 6: T1–10-T2, lane 7: T1–20-T2, lane 8: Caliper-14, lane 9: Caliper-17, lane 10: Caliper-20, lane 11: Caliper-23. (e) 3D structural representation of the TCR-CD3 dimer and Caliper-14/17/20/23. The TCR-CD3 complex structure was obtained from the Protein Data Bank (PDB ID: 6JXR). (f) Fluorescence kinetic curves of Caliper-14/17/20/23 interacting with plasma from naive mice (*n* = 3). (g) The Δ*F*_FAM_/Δ*F*_ROX_ ratio corresponding to the fluorescence response upon interaction with plasma from naive mice. (h) FI ratios in plasma from naive, autograft, and allograft mice measured using Caliper-14/17/20/23 (*n* = 6). Statistical significance was determined using two-way ANOVA followed by Tukey's multiple comparisons test. Violin plots of FI ratios in plasma from autograft (i) and allograft (j) mice post-transplantation (days 0, 3, 7, 10, and 15), measured using Caliper-17 (*n* = 5). Statistical significance was assessed using a one-way ANOVA followed by Tukey’s multiple comparisons test. *p < 0.05; **p < 0.01; ***p < 0.001, ns = no significance. Data are presented as mean ± s.d.

Considering that optimal aptamer spacing is essential for the simultaneous engagement of dimeric TCR-CD3 complexes, we designed four caliper probes and validated their assembly by PAGE analysis (Figure 3d). Structural modeling of the TCR-CD3 dimer and the four caliper probes was performed using UCSF ChimeraX.^33^ As shown in Figure 3e, the distance between the two opposing CD3ε subunits (colored green) is measured at 148 Å, which is consistent with previous cryo-EM results.^34^ Upon binding to CD3ε, the aptamer segments of the caliper probes unwind. The distances between the terminal bases of the two aptamers are calculated to be 142, 170, 204, and 232 Å, respectively, and those probes are correspondingly denoted as Caliper-14, Caliper-17, Caliper-20, and Caliper-23. When the terminal aptamer binds to one CD3ε, the relative position of the central aptamer with respect to the second CD3ε is evaluated. Caliper-14 experiences substantial steric hindrance when attempting to bind the second CD3ε, whereas for Caliper-20 and Caliper-23, the central aptamer is positioned too far from the second CD3ε to form an efficient interaction. Among them, Caliper-17 presents a geometrically favorable configuration, wherein the spacing of the two aptamer strands matches well with the positions of the two CD3ε subunits in the TCR-CD3 dimer, therefore minimizing steric clashes. Importantly, this static model provides a structural explanation rather than direct proof of an exact distance match. In practice, engagement process is collectively influenced by probe flexibility, steric constraints, receptor density, and assembly differences. To empirically evaluate their actual performance, fluorescence kinetic measurements in naive mouse plasma were conducted, which showed a pronounced increase in the ROX signal and the lowest Δ*F*_FAM_/Δ*F*_ROX_ ratio for Caliper-14 (Figure 3f,g). From Caliper-17 to Caliper-23, this ratio gradually declined, suggesting that 170 Å represents a highly effective aptamer spacing for the engagement of both CD3ε subunits.

Subsequently, these caliper probes were applied to analyze plasma samples from naive, autograft, and allograft mice. The individual FAM and ROX fluorescence intensities showed no significant differences among the groups (Figure S15). This lack of difference may be attributed to the fact overall EV abundance can fluctuate based on factors such as age, sex, health status, and sample handling conditions.^35,36^ Benefiting from the ratiometric measurement approach of the caliper probes, the FI ratio remained stable (variation within ±3%) when the plasma input volume was varied from 5 to 15 μL (Figure S16). This robustness effectively eliminates the interference caused by variations in EV abundance, enabling clear comparison of TCR–CD3 oligomerization states across individuals. Caliper-17, Caliper-20, and Caliper-23 showed elevated FI ratios in the allograft group while maintaining similar values in the naive and autograft groups, with Caliper-17 exhibiting the most significant detection difference (Figure 3h). In contrast, Caliper-14 exhibited no significant differences in FI ratios among the three groups, indicating its inability to differentiate TCR-CD3 oligomeric states. To further correlate TCR-CD3 oligomeric EVs with rejection progression, plasma samples were collected on days 3, 7, 10, and 15 post-transplantations. The FI ratios in the autograft group remained stable over time (Figure 3i), whereas the allograft group displayed a progressive upward trend, peaking at day 15, which coincided with full graft rejection (Figure 3j). These findings identify TCR-CD3 oligomeric EVs as potential biomarkers for T cell-mediated rejection and underscore the broad potential of distance-tuned caliper probes for detecting protein oligomerization.

### Discrimination between Rejection and Infection Using Caliper-17

To evaluate the diagnostic specificity of Caliper-17 in distinguishing transplant rejection from infection, we established a cecal ligation and puncture (CLP) infection model (Figure S17). Widely regarded as the gold standard for experimental sepsis, this model replicates the progression and systemic manifestations of human polymicrobial infection by initiating an intra-abdominal infection that elicits a robust inflammatory response.^37^ To simulate a clinically relevant scenario where infection and graft rejection coexist, we developed a dual disease model (Rej-Inf group) by performing CLP on day 15 following allogeneic skin transplantation (Figure 4a). Histological examination of Rej-Inf grafts revealed inflammatory and T cell infiltration comparable to the Rejection group alone (Figure 4b), suggesting that T cell-mediated rejection persisted. However, quantification of plasma cytokines including interleukin-6 (IL-6), tumor necrosis factor-α (TNF-α), and monocyte chemoattractant protein-1 (MCP-1), which are key mediators of immune responses to fight off infection and inflammation,^38,39^ showed that their levels remained unchanged in the Rejection group compared to those in Naive mice, but were significantly elevated in both the Infection and Rej-Inf groups (Figures 4c and S18), indicating a dominant role of infection in driving systemic inflammatory activation. Furthermore, flow cytometric analysis revealed that circulating CD3+ T cells were significantly reduced in infection-bearing mice (Figures 4d and S19), likely reflecting the acute infection-induced thymic atrophy.^40^ In contrast, the proportion of peripheral CD8+ GzmB + cytotoxic T cells, whose granzyme B expression mediates target cell apoptosis during graft injury,^41^ was significantly elevated in the Rejection group (Figures 4e and S20). Although this population also increased in the Rej-Inf group, the magnitude was notably attenuated, suggesting that infection may suppress cytotoxic T cell responses during ongoing rejection. Together, these findings imply that T cell-mediated rejection may be largely confined to the graft microenvironment, and that the systemic cytokine surge and immune alterations induced by infection can mask or interfere with the detection of concurrent rejection processes.

**Figure 4:**
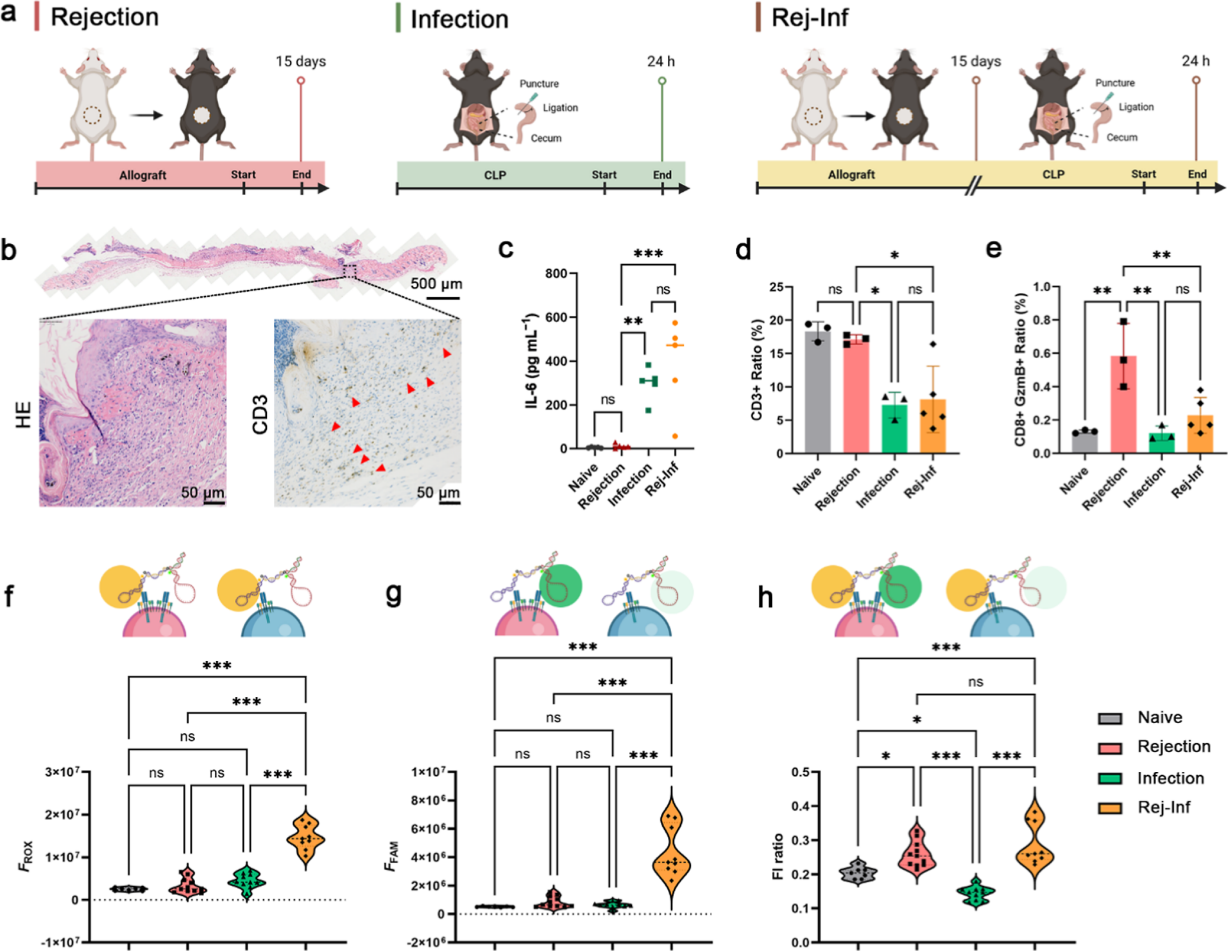
Distinction of allograft rejection from infection using Caliper-17. (a) Schematic illustration of experimental mouse models representing the Rejection, Infection, and Rej-Inf groups. The diagram outlines the operation strategies applied to each group, including allogeneic transplantation, CLP-induced infection, and the corresponding time course. (b) H & E staining of full-thickness skin from the Rej-Inf group. The junction between the grafted and host skin is marked by a dashed box. Left: magnified H & E images of the boxed region. Right: magnified CD3 IHC staining of the same area. (c) Plasma concentrations of IL-6 measured by ELISA. (d) Flow cytometric analysis of the positive ratio of CD3+ T cells (d) and CD8+ GzmB + cytotoxic T cells (e) in peripheral blood (mean ± s.d., *n* = 5 for the Rej-Inf group, *n* = 3 for other groups). Violin plots showing *F*_ROX_ (f), *F*_FAM_ (g), and FI ratios (h) in plasma samples from Naive, Rejection, Infection, and Rej-Inf groups, as detected by Caliper-17. Statistical significance was assessed using one-way ANOVA followed by Tukey’s multiple comparisons test (ns = no significance; *p < 0.05; **p < 0.01; ***p < 0.001).

To overcome this diagnostic challenge, we evaluated the capacity of Caliper-17 to accurately differentiate between these conditions. The ROX fluorescence signal of the caliper probe, which is indicative of total CD3+ EV abundance, was elevated in both the Infection and Rej-Inf groups (Figure 4f). This suggests an increased release of T cell-derived EVs under infectious conditions, a phenomena similarly reported in Epstein–Barr virus infection.^42^ In contrast, the FAM fluorescence signal, which reflects the presence of TCR-CD3 dimeric complexes, was reduced in the Infection group but remained elevated in the Rej-Inf group (Figure 4g). Upon normalization (Figure 4h), the FI ratio was significantly increased in the Rejection group (0.26 ± 0.04) and decreased in the Infection group (0.14 ± 0.02) relative to Naive controls (0.20 ± 0.02). Notably, the Rej-Inf group exhibited an FI ratio (0.29 ± 0.06) comparable to that of the Rejection group. These results demonstrated that Caliper-17 accurately distinguishes T cell-mediated rejection from infection, even in case where both conditions coexist.

### RNA Sequencing Analysis of Rejection, Infection, and Rej-Inf Mouse Models

Given the distinct immunopathological mechanisms underlying allograft rejection and infection, as well as the lack of prior characterization of a concurrent Rej-Inf model, we conducted RNA sequencing on spleen tissues from the Naive, Rejection, Infection, and ref-Inf groups. As the largest secondary lymphoid organ, the spleen plays a central role in systemic immune surveillance and integrates signals from both innate and adaptive immune cells.^43^ Thus, transcriptomic profiling of splenic tissue provides a comprehensive overview of systemic immunological activity. Principal component analysis (PCA) revealed a clear separation among the Naive, Rejection, and Infection groups, reflecting their distinct immune signatures (Figure 5a). The Rej-Inf group clustered closer to the Infection group but was shifted toward the Rejection group, suggesting a mixed immune state that incorporates features of both conditions.

**Figure 5:**
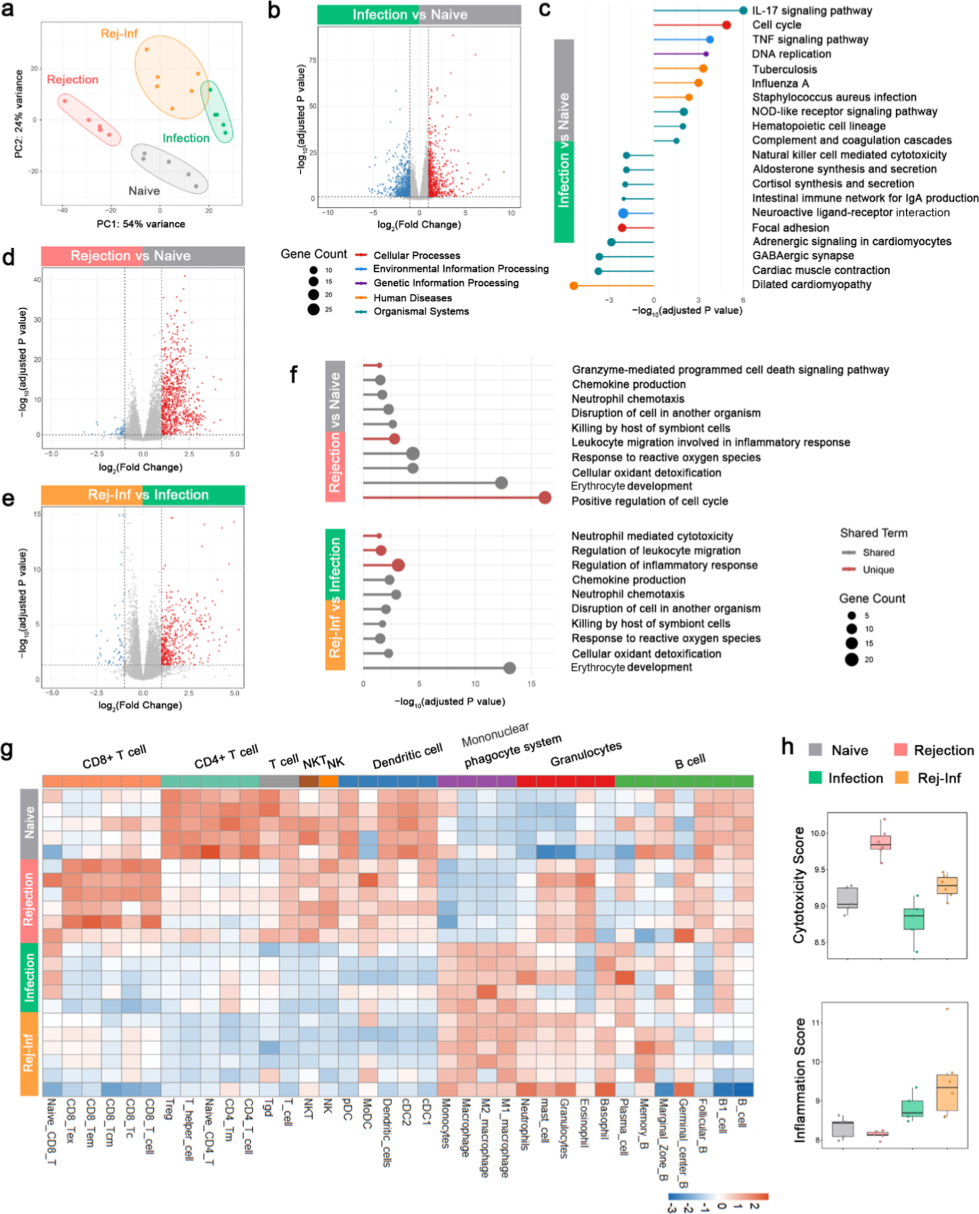
RNA sequencing analysis of spleens from mice in the Naive, Rejection, Infection, and Rej-Inf groups. (a) PCA plot showing distinct clustering of gene expression profiles across the four groups (*n* = 22). (b) Volcano plot illustrating DEGs between the Infection and Naive groups. Red indicates upregulated genes; blue indicated downregulated genes. (c) KEGG pathway enrichment analysis identifying significantly upregulated and downregulated pathways in the Infection group compared to the Naive group. Volcano plots showing DEGs between the Rejection and Naive groups (d), and between the Rej-Inf and Infection groups (e). (f) GO enrichment analysis of upregulated genes in the Rejection group (vs Naive) and in the Rej-Inf group (vs Infection), highlighting shared biological processes. (g) Heatmaps showing enrichment scores of immune cell signature genes across the four groups. (h) Boxplots of cytotoxicity and inflammation scores based on the expression of signature genes (cytotoxicity: GZMB, GZMA, PRF1, NKG7, KLRK1; inflammation: TNF, IL1B, CXCL10, CXCL9, IL6).

Compared to Naive controls, the Infection group exhibited 694 upregulated and 893 downregulated differentially expressed genes (DEGs) (Figure 5b). Kyoto Encyclopedia of Genes and Genomes (KEGG) pathway enrichment analysis identified a significant upregulation of the IL-17, TNF, and Toll-like receptor signaling pathways (Figure 5c). These pathways are central to the host defense against pathogens, driving an inflammatory cascade, mediating the recruitment and activation of innate immune cells, and triggering cell death.^44–46^ The enrichment of pathways related to specific infections (e.g., tuberculosis, influenza A, and *Staphylococcus aureus* infection) further underscored the activation of antimicrobial responses. Conversely, downregulated pathways in the Infection group involved natural killer (NK) cell-mediated cytotoxicity, intestinal mucosal immunity, neuroimmune regulation, and cardiovascular function, reflecting concurrent immunosuppressive and tissue-damaging effects. These findings highlight the dual nature of infection-induced immune alterations: systemic hyperinflammation coupled with local or functional immune dysregulation.

In the Rejection group, 839 genes were upregulated and 41 were downregulated relative to the Naive group (Figure 5d). Upon concurrent rejection and infection, the Rej-Inf group showed 434 upregulated and 49 downregulated DEGs compared to the Infection group (Figure 5e). Gene Ontology (GO) enrichment analysis revealed that both the Rejection and Rej-Inf groups shared an upregulation of cytotoxicity-related biological processes, including “disruption of cell in another organism” and “killing by host of symbiont cells” (Figure 5f). These transcriptomic profiles highlight a shared reliance on cytotoxic effector mechanisms targeting foreign or allogeneic cells in both allograft rejection and the combined Rej-Inf condition.

To further dissect immune cell alterations across the models, we employed ImmunCellAI-mouse tool to infer the abundance of 35 immune cell subtypes from the gene expression data.^47^ As shown in Figures 5g and S21, CD8+ T cells, particularly CD8+ exhausted T cells (*T*_ex_), CD8+ effector memory T cells (*T*_em_), and CD8+ cytotoxic T cells (*T*_c_), were significantly increased in the Rejection group relative to Naive controls, and were also elevated in the Rej-Inf group compared to the Infection group. In contrast, the monocyte-macrophage system was markedly expanded in the Infection and Rej-Inf groups (Figure S22). Cytotoxicity and inflammation scores, derived from the average expression of curated gene signatures, revealed that the Rejection and Rej-Inf groups exhibited elevated cytotoxicity, while the Infection and Rej-Inf groups showed heightened inflammation activity (Figure 5h). This divergence in systemic immune architecture is consistent with our EV measurements: allograft rejection, driven primarily by activated CD8+ cytotoxic T cells, is associated with a pronounced increase in oligomeric TCR-CD3 on T cell-derived EVs and a higher FI ratio, whereas infection, governed predominantly by monocyte-macrophage system, corresponds to a much lower TCR-CD3 oligomeric signal.

Accordingly, we infer that while the Caliper-17 assay can detect circulating EV-associated TCR-CD3 oligomerization originating from a broader range of T cell populations, the elevated signals observed during rejection likely reflect a significant contribution from activated CD8+ cytotoxic T cells. The expansion of CD8+ cytotoxic T cells in both the Rejection and Rej-Inf groups, together with our in vitro coculture results demonstrating that TCR-CD3 oligomeric EVs arise from immunological synapses of activated CD8+ T cells, provides a mechanistic basis for the elevated biomarker signal in the rejection cohort. In this context, persistent alloantigen exposure drives strong and sustained TCR engagement, leading to enhanced synapse formation and remodeling, which in turn results in elevated release of TCR-CD3 oligomeric EVs. By quantifying these EVs, the Caliper-17 assay enables monitoring of overall T cell activation, including prominent CD8+ effector responses triggered by alloantigen recognition.

### Clinical Utility of Caliper-17 to Discriminate Rejection from Infection

To explore the clinical potential of TCR-CD3 oligomeric EVs as biomarkers for rejection, we collected plasma samples from a total of 34 liver or kidney transplant recipients. These included 17 patients in the rejection group, 10 in the infection group, and 7 in the concurrent Rej-Inf group. Tissue biopsy and microbiological identification were used as the gold standards for diagnosing rejection and infection, respectively (Table S2 and Figure 6a). Distinguishing between rejection and infection prior to invasive biopsy and time-consuming microbiological identification is a major diagnostic dilemma, making it exceptionally challenging to guide timely medication. As shown in Figure 6b, the FI ratios measured by the Caliper-7 assay stabilized approximately 2 h after incubation, offering a rapid detection strategy. The FI ratios were significantly higher in patients with rejection (0.77 ± 0.24) than in those with infection (0.50 ± 0.20) and remained elevated in the Rej-Inf cohort (1.00 ± 0.39) (Figure 6c). Furthermore, to evaluate the overall diagnostic performance of the Caliper-17 assay, we performed a receiver operating characteristic (ROC) curve analysis on our clinical cohorts (Figure 6d). The assay demonstrated strong diagnostic potential with an Area Under the Curve (AUC) of 0.85 (95% confidence interval (CI): 0.72–0.99; *p* = 0.001). Using the Youden index to determine the optimal threshold of FI ratio, a cutoff value of 0.69 yielded a sensitivity of 71% (95% CI: 51%–85%) and a specificity of 90% (95% CI: 60%–99%) in distinguishing allograft rejection from infection. A positive result at this threshold could potentially suggest the presence of T cell-mediated rejection and inform more aggressive antirejection treatment, whereas a negative result might make rejection less likely and support prioritizing infection-focused management and avoiding unnecessary increases in immunosuppression. The accuracy for our clinical testing of 34 samples is 76%. Specifically, at the 0.69 cutoff, the assay yielded 17 true positives, 1 false positive, 9 true negatives, and 7 false negatives (detailed in the confusion matrix in Table S3). Therefore, this approach holds promise to meaningfully reduce diagnostic uncertainty and assist in guiding more timely and appropriate therapeutic decisions in complex post-transplant settings. Nevertheless, despite these encouraging findings, the current cohort is relatively small, and the diagnostic cutoff was derived from the same data set. Therefore, validation in larger, independent, multicenter cohorts is necessary before this threshold can be reliably implemented in clinical practice.

**Figure 6:**
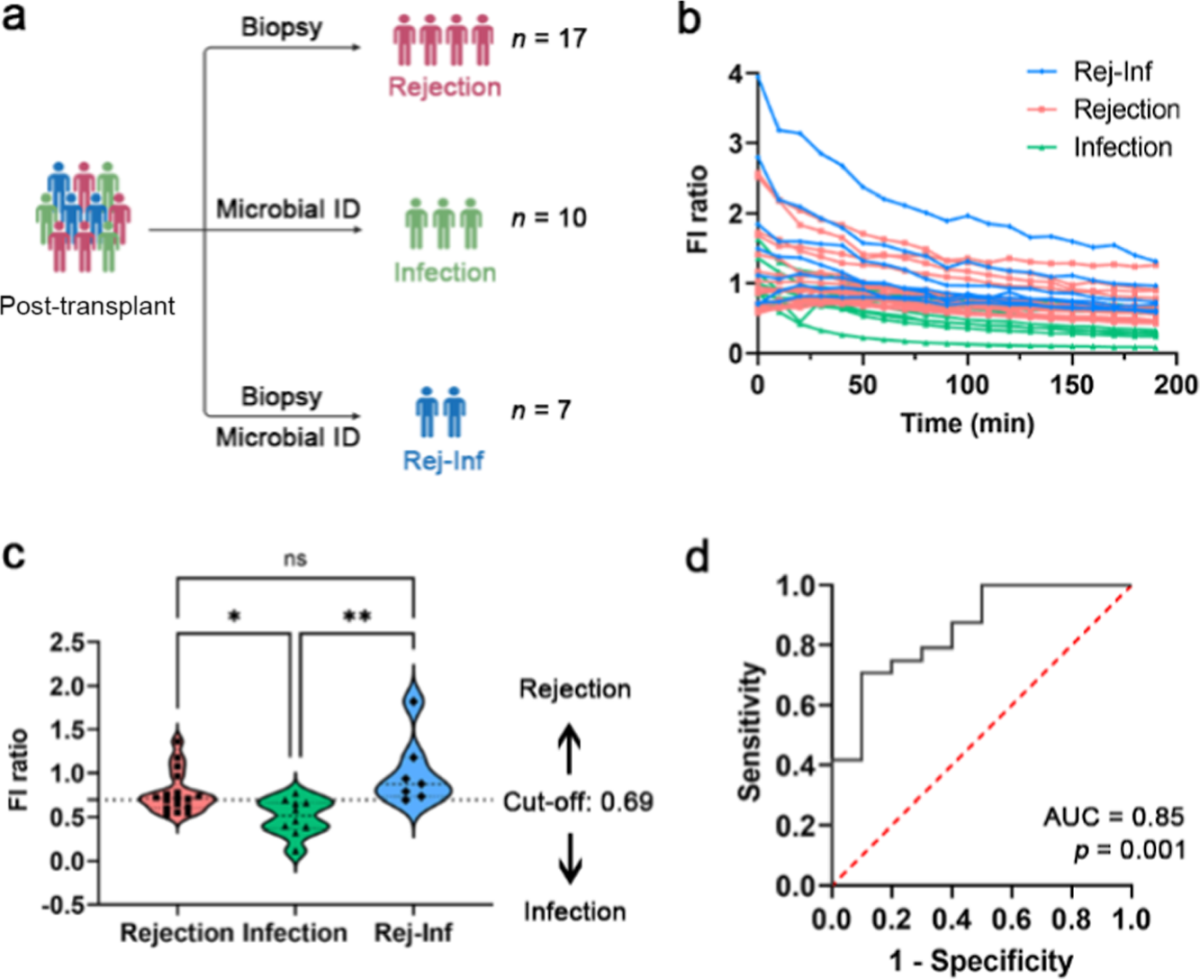
Clinical utility of TCR-CD3 oligomeric EVs as biomarkers for acute cellular rejection. (a) Composition of the clinical cohort comprising post-transplant patients with rejection (*n* = 17), infection (*n* = 10), or concurrent rejection and infection (Rej-Inf, *n* = 7), as determined by tissue biopsy and microbial identification (ID). (b) Fluorescence kinetic curves of Caliper-17 interacting with clinical plasma samples. (c) FI ratios of clinical plasma samples detected by the Caliper-17 assay. The dashed line indicates the optimal cutoff value determined using the Youden index. Statistical significance was assessed using one-way ANOVA followed by Tukey’s multiple comparisons test (ns = no significance; *p < 0.05; **p < 0.01). (d) ROC curve analysis for distinguishing allograft rejection from infection.

Given the critical importance of reproducibility and stability for clinical biomarkers, we evaluated both the intra- and interassay precision of the Caliper-17 assay using clinical samples among three groups. Within a single run, the assay demonstrated superior intra-assay precision, yielding an average coefficient of variation (CV) of 4.27% (range: 1.33%–12.10%) (Table S4). To further test the same clinical plasma samples across different storage conditions, three independent assays were run under distinct conditions: freshly isolated, stored at 4 °C overnight, and subjected to a single freeze–thaw cycle from −80 °C to room temperature. The calculated interassay CV across these three diverse conditions was 9.13% (range: 5.50%–13.06%), and there was no statistically significant difference in the FI ratio among the variable preanalyzed conditions (Figure S23). Overall, the Caliper-17 assay demonstrated good reproducibility and robustness, performing well within the acceptable clinical range for potential clinical translation.

## Discussion

EVs have been widely studied as potential biomarkers for transplant rejection and infection, with various molecular components been explored, including internal proteins, mRNAs, microRNAs, and surface proteins.^48,49^ For instance, two previous studies demonstrated the utility of urinary EVs in detecting T cell-mediated rejection by analyzing surface CD3 or internal proteins, such as tetraspanin-1 and hemopexin.^50,51^ While promising for kidney transplantation, these approaches may have limited applicability in other types of organ transplants. Rather than merely measuring changes in EV abundance or overall protein expression, our strategy gains insight into the activation status of the parent T cells based on the oligomeric state of their surface TCR-CD3 complexes. This mechanistic focus offers broader potential for application across diverse organ transplant settings.

Proteins rarely function in isolation. Protein self-association, such as homodimer and oligomer formation, is a ubiquitous phenomenon in biological systems that contributes to protein stability, activity regulation, and functional complexity.^52,53^ Many techniques are available to study protein oligomerization in cells, including chemical cross-linking, fluorescence correlation spectroscopy, and Förster resonance energy transfer.^54,55^ Fluorescent signals generated from interacting aptamer pairs targeting distinct proteins can provide indirect evidence of protein colocalization or interaction.^56,57^ However, when targeting identical proteins, aptamer binding often induces or relies on multivalent interactions that lead to aggregation, thereby limiting the ability to resolve their spatial distribution.^58–60^ Our caliper probe addresses this critical limitation by precisely tuning the affinity, spacing, and arrangement of the aptamer switch probes, thereby allowing sequential binding to the monomeric and oligomeric states of the same protein. Once customized, this modular platform can be broadly extended to investigate other oligomerization-dependent receptors, such as B cell receptor (BCR),^61^ chimeric antigen receptor (CAR),^62^ epidermal growth factor receptor (EGFR),^63^ and G protein-coupled receptors (GPCRs)^64^ across various contexts including immune activation, cancer proliferation, and drug susceptibility.

Despite these promising results, our study has several limitations. First, because our current assay targets CD3, it detects circulating EV-associated TCR-CD3 oligomerization from a broader range of T cell populations, inherently capturing a global pool of EVs derived from all CD3-expressing cells. This includes both CD4+ and CD8+ T cell subsets, as well as minor populations such as NK T and γδ T cells. Although RNA sequencing in our mouse models revealed that elevated levels of TCR-CD3 oligomeric EVs correlate with CD8+ cytotoxic T cell activation, the precise secretion pathways and immunological function of these EVs remain incomplete understood. Future iterations employing subset-specific probes (e.g., targeting CD4 or CD8) will be required to elucidate the comprehensive molecular mechanisms governing these EVs. Second, our clinical validation was conducted in a relatively small cohort, primarily involving liver and kidney transplant recipients experiencing rejection or sepsis. To demonstrate broader clinical utility, future studies must incorporate larger, multicenter cohorts encompassing diverse patient populations, such as those undergoing heart, lung, or islet transplantation. Finally, the potential impacts of immunosuppressive drugs on TCR-CD3 oligomerization were not exhaustively explored. Although we validated the biomarker in clinical samples from patients treated with tacrolimus, cyclosporine, or sirolimus, different immunosuppressants may modulate T cells through distinct mechanisms.^9^ Further characterization is essential to thoroughly assess their confounding effects on CD3+ EV profiles.

With continued optimization and large-scale validation, we anticipate that this technology will translate into a practical and cost-effective diagnostic tool for clinical use. The caliper probe can be integrated into microvolume plasma separation platforms and coupled with smartphone-based optical readouts, enabling real-time, point-of-care monitoring of patient immune status post-transplantation. As a minimally invasive diagnostic approach, it holds significant potential to refine current detection methods for rejection and infection, reduce patient discomfort, and facilitate the timely adjustment of immunosuppressive regimens, ultimately serving as a safe, convenient, and robust solution for personalized transplant management.

## Conclusion

Rejection and infection are among the leading causes of graft failure and mortality following organ transplantation. Distinguishing between these two complications based solely on clinical manifestations and conventional biomarkers remains challenging, often resulting in delayed or inappropriate treatment. In this study, we introduce TCR-CD3 oligomeric EVs as novel biomarkers reflecting allogeneic T cell activation, and we develop a ratiometric caliper probe to specifically detect the oligomeric state of TCR-CD3 complexes on circulating EVs. Through comprehensive analyses in in vitro and in vivo models, as well as in clinical samples, our method enabled the quantification of the TCR-CD3 oligomer-to-monomer ratio, which was found to be elevated during rejection and reduced during infection. This approach offers a minimally invasive method for diagnostically distinguishing between rejection and infection, effectively overcoming the limitations posed by overlapping clinical presentations.

## Supplementary Material



## Notes

Detailed experimental procedures, materials and methods, calculation of binding abilities, demographic characteristics, confusion matrix, and reproducibility of clinical samples, nucleic acid sequences, *K*_d_ of TA14 and TA18, EV-dependent fluorescence responses of TA10 and TA20, fluorescent background and specificity of Caliper-17, size characterization of plasma EVs, kinetic analyses in plasma, identification of BMDCs and CD8+ T cells, fluorescence intensities and *n*FCM characterization of EVs, H & E staining of skin tissues, flow cytometric analysis of CD4, CD8, CD3, and GzmB, fluorescence intensities in mouse plasma, FI ratios across different plasma inputs, image of the CLP model, cytokine profiling by ELISA, immune cell subset analyses, and assessment of assay robustness (PDF)

## References

[1] Agostini C., Buccianti S., Risaliti M., Fortuna L., Tirloni L., Tucci R., Bartolini I., Grazi G. L. (2023). Complications in
Post-Liver Transplant Patients. J. Clin. Med..

[2] Higdon L. E., Tan J. C., Maltzman J. S. (2023). Infection, Rejection,
and the Connection. Transplantation.

[3] Power A., Baez Hernandez N., Dipchand A. I. (2022). Rejection Surveillance in Pediatric
Heart Transplant Recipients: Critical Reflection on the Role of Frequent
and Long-Term Routine Surveillance Endomyocardial Biopsies and Comprehensive
Review of Non-Invasive Rejection Screening Tools. Pediatr. Transplant..

[4] Simon S., Kaiser M. S., Bachmann M., Krause G., Gottlieb J. (2026). Point-of-Care
Testing by Multiplex-PCR in Different Compartments in Suspected Lower
Respiratory Tract Infection After Lung Transplantation-Results of
a Prospective Study. Transpl. Infect. Dis..

[5] Song Y., Wang Y., Wang W., Xie Y., Zhang J., Liu J., Jin Q., Wu W., Li H., Wang J., Zhang L., Yang Y., Gao T., Xie M. (2025). Advancements
in Noninvasive Techniques for Transplant Rejection: From Biomarker
Detection to Molecular Imaging. J. Transl. Med..

[6] He R. R., Yue G. L., Dong M. L., Wang J. Q., Cheng C. (2024). Sepsis Biomarkers:
Advancements and Clinical Applications—A Narrative Review. Int. J. Mol. Sci..

[7] Tharmaraj D., Mulley W. R., Dendle C. (2024). Current and
Emerging Tools for Simultaneous
Assessment of Infection and Rejection Risk in Transplantation. Front. Immunol..

[8] Li Q., Lan P. (2023). Activation of Immune
Signals During Organ Transplantation. Signal
Transduct. Target. Ther..

[9] Elalouf A. (2023). Infections
After Organ Transplantation and Immune Response. Transpl. Immunol.

[10] Rodrigo E., López-Hoyos M., Corral M., Fábrega E., Fernández-Fresnedo G., San Segundo D., Piñera C., Arias M. (2012). ImmuKnow as a Diagnostic
Tool for
Predicting Infection and Acute Rejection in Adult Liver Transplant
Recipients: A Systematic Review and Meta-Analysis. Liver Transpl.

[11] Fernandez
Valledor A., Moeller C. M., Rubinstein G., Oren D., Rahman S., Baranowska J., Lee C., Lotan D., DeFilippis E. M., Theodoropoulos K., Bae D., Oh K., Clerkin K., Fried J., Raikhelkar J., Latif F., Uriel N., Sayer G. (2024). Is There A Correlation
Between a High Immuknow Level and Increased Rates of Rejection? Insights
from Molecular Microscope (MMDx). J. Heart Lung
Transplant..

[12] Ling X., Xiong J., Liang W., Schroder P. M., Wu L., Ju W., Kong Y., Shang Y., Guo Z., He X. (2012). Can Immune
Cell Function Assay Identify Patients at Risk of Infection or Rejection?
A Meta-Analysis. Transplantation.

[13] Minguet S., Swamy M., Alarcón B., Luescher I. F., Schamel W. W. (2007). Full Activation
of the T Cell Receptor Requires Both Clustering and Conformational
Changes at CD3. Immunity.

[14] van
der Merwe P. A., Dushek O. (2011). Mechanisms for T Cell Receptor Triggering. Nat. Rev. Immunol..

[15] Sun Y., Yan L., Sun J., Xiao M., Lai W., Song G., Li L., Fan C., Pei H. (2022). Nanoscale Organization of Two-Dimensional
Multimeric pMHC Reagents with DNA Origami for CD8+ T Cell Detection. Nat. Commun..

[16] Sun Y., Sun J., Xiao M., Lai W., Li L., Fan C., Pei H. (2022). DNA Origami–Based
Artificial Antigen-Presenting Cells for
Adoptive T Cell Therapy. Sci. Adv..

[17] Pageon S. V., Tabarin T., Yamamoto Y., Ma Y., Nicovich P. R., Bridgeman J. S., Cohnen A., Benzing C., Gao Y., Crowther M. D., Tungatt K., Dolton G., Sewell A. K., Price D. A., Acuto O., Parton R. G., Gooding J. J., Rossy J., Rossjohn J., Gaus K. (2016). Functional Role of
T-Cell Receptor Nanoclusters in Signal Initiation and Antigen Discrimination. Proc. Natl. Acad. Sci. U.S.A..

[18] Choudhuri K., Llodrá J., Roth E. W., Tsai J., Gordo S., Wucherpfennig K. W., Kam L. C., Stokes D. L., Dustin M. L. (2014). Polarized
Release of T-Cell-Receptor-Enriched Microvesicles at the Immunological
Synapse. Nature.

[19] Blanchard N., Lankar D., Faure F., Regnault A., Dumont C., Raposo G., Hivroz C. (2002). TCR Activation of Human
T Cells Induces
the Production of Exosomes Bearing the TCR/CD3/Zeta Complex. J. Immunol..

[20] Stinchcombe J. C., Asano Y., Kaufman C. J. G., Böhlig K., Peddie C. J., Collinson L. M., Nadler A., Griffiths G. M. (2023). Ectocytosis
Renders T Cell Receptor Signaling Self-Limiting at the Immune Synapse. Science.

[21] Yin W., Chen H., Cai J., Huang X., Zhang L., Xu Y., Zheng J., Liu S.-Y., Zou X., Dai Z., Yang Y. (2023). Isolation of Structurally Heterogeneous TCR-CD3 Extracellular Vesicle
Subpopulations Using Caliper Strategy. Angew.
Chem. Int. Ed..

[22] Hendrix A., Lippens L., Pinheiro C., Théry C., Martin-Jaular L., Lötvall J., Lässer C., Hill A. F., Witwer K. W. (2023). Extracellular Vesicle Analysis. Nat. Rev. Methods Primers.

[23] Tang Z., Mallikaratchy P., Yang R., Kim Y., Zhu Z., Wang H., Tan W. (2008). Aptamer Switch Probe Based on Intramolecular
Displacement. J. Am. Chem. Soc..

[24] Hariri A. A., Cartwright A. P., Dory C., Gidi Y., Yee S., Thompson I. A. P., Fu K. X., Yang K., Wu D., Maganzini N., Feagin T., Young B. E., Afshar B. H., Eisenstein M., Digonnet M. J. F., Vuckovic J., Soh H. T. (2024). Modular
Aptamer Switches for the Continuous Optical Detection of Small-Molecule
Analytes in Complex Media. Adv. Mater..

[25] Schamel W. W., Arechaga I., Risueño R. M., van Santen H. M., Cabezas P., Risco C., Valpuesta J. M., Alarcón B. (2005). Coexistence of Multivalent and Monovalent TCRs Explains
High Sensitivity and Wide Range of Response. J. Exp. Med..

[26] Chao Z., Mei Q., Yang C., Luo J., Liu P., Peng H., Guo X., Yin Z., Li L., Wang Z. (2025). Immunological Synapse:
Structures, Molecular Mechanisms, and Therapeutic Implications in
Disease. Signal Transduct. Target. Ther..

[27] Strazza M., Song R., Hiner S., Mor A. (2024). Changing the Location
of Proteins on the Cell Surface Is a Promising Strategy for Modulating
T Cell Functions. Immunology.

[28] Ruiz-Navarro J., Calvo V., Izquierdo M. (2024). Extracellular Vesicles and Microvilli
in the Immune Synapse. Front. Immunol..

[29] Bálint S. ˇ., Müller S., Fischer R., Kessler B. M., Harkiolaki M., Valitutti S., Dustin M. L. (2020). Supramolecular Attack
Particles Are Autonomous Killing Entities Released from Cytotoxic
T Cells. Science.

[30] Kim H.-R., Mun Y., Lee K.-S., Park Y.-J., Park J.-S., Park J.-H., Jeon B.-N., Kim C.-H., Jun Y., Hyun Y.-M., Kim M., Lee S.-M., Park C.-S., Im S.-H., Jun C.-D. (2018). T Cell
Microvilli Constitute Immunological Synaptosomes That Carry Messages
to Antigen-Presenting Cells. Nat. Commun..

[31] Cheng C. H., Lee C. F., Fryer M., Furtmüller G. J., Oh B., Powell J. D., Brandacher G. (2017). Murine Full-Thickness
Skin Transplantation. J. Vis. Exp..

[32] Shenoy K. V., Solomides C., Cordova F., Rogers T. J., Ciccolella D., Criner G. J. (2012). Low CD4/CD8 Ratio in Bronchus-Associated Lymphoid Tissue
Is Associated with Lung Allograft Rejection. J. Transplant..

[33] Meng E. C., Goddard T. D., Pettersen E. F., Couch G. S., Pearson Z. J., Morris J. H., Ferrin T. E. (2023). UCSF ChimeraX:
Tools for Structure
Building and Analysis. Protein Sci..

[34] Birnbaum M. E., Berry R., Hsiao Y. S., Chen Z., Shingu-Vazquez M. A., Yu X., Waghray D., Fischer S., McCluskey J., Rossjohn J., Walz T., Garcia K. C. (2014). Molecular Architecture
of the αβ T Cell Receptor–CD3 Complex. Proc. Natl. Acad. Sci. U. S. A..

[35] Bæk R., Søndergaard E. K., Varming K., Jørgensen M. M. (2016). The Impact
of Various Preanalytical Treatments on the Phenotype of Small Extracellular
Vesicles in Blood Analyzed by Protein Microarray. J. Immunol. Methods.

[36] Noren
Hooten N., Byappanahalli A. M., Vannoy M., Omoniyi V., Evans M. K. (2022). Influences of Age, Race, and Sex on Extracellular Vesicle
Characteristics. Theranostics.

[37] Kannan S. K., Kim C. Y., Heidarian M., Berton R. R., Jensen I. J., Griffith T. S., Badovinac V. P. (2024). Mouse Models
of Sepsis. Curr. Protoc..

[38] Al-Qahtani A. A., Alhamlan F. S., Al-Qahtani A. A. (2024). Pro-Inflammatory and Anti-Inflammatory
Interleukins in Infectious Diseases: A Comprehensive Review. Trop. Med. Infect. Dis..

[39] Singh S., Anshita D., Ravichandiran V. (2021). MCP-1: Function,
Regulation, and
Involvement in Disease. Int. Immunopharmacol..

[40] Velardi E., Tsai J. J., van den Brink M. R. M. (2021). T Cell
Regeneration After Immunological
Injury. Nat. Rev. Immunol..

[41] Li X., Chen G., Wu K., Zheng H., Tian Z., Xu Z., Zhao W., Weng J., Min Y. (2024). Imaging and Monitoring
of Granzyme B in the Immune Response. Wiley
Interdiscip. Rev. Nanomed. Nanobiotechnol..

[42] Oba R., Isomura M., Igarashi A., Nagata K. (2019). Circulating CD3­(+)­HLA-DR­(+)
Extracellular Vesicles as a Marker for Th1/Tc1-Type Immune Responses. J. Immunol. Res..

[43] Akhand, A. A. ; Ahsan, N. Cells and Organs of the Immune System. In Immunology for Dentistry, 2023; pp 1–12.

[44] Huangfu L., Li R., Huang Y., Wang S. (2023). The IL-17 Family in Diseases: From
Bench to Bedside. Signal Transduct. Target.
Ther..

[45] Huyghe J., Priem D., Bertrand M. J. M. (2023). Cell
Death Checkpoints in the TNF
Pathway. Trends Immunol..

[46] Fisch D., Zhang T., Sun H., Ma W., Tan Y., Gygi S. P., Higgins D. E., Kagan J. C. (2024). Molecular Definition
of the Endogenous Toll-Like Receptor Signalling Pathways. Nature.

[47] Miao Y. R., Xia M., Luo M., Luo T., Yang M., Guo A. Y. (2022). ImmuCellAI-Mouse:
A Tool for Comprehensive Prediction of Mouse Immune Cell Abundance
and Immune Microenvironment Depiction. Bioinformatics.

[48] Thongwitokomarn H., Noppakun K., Chaiwarith R., Chattipakorn S. C., Chattipakorn N. (2024). Extracellular Vesicles as Potential
Diagnostic Markers
for Kidney Allograft Rejection. Clin. Transplant..

[49] A J., S S.
S., K S., T S.
M. (2023). Extracellular Vesicles
in Bacterial and Fungal Diseases: Pathogenesis to Diagnostic Biomarkers. Virulence.

[50] Lim J. H., Lee C. H., Kim K. Y., Jung H. Y., Choi J. Y., Cho J. H., Park S. H., Kim Y. L., Baek M. C., Park J. B., Kim Y. H., Chung B. H., Lee S. H., Kim C. D. (2018). Novel Urinary Exosomal
Biomarkers of Acute T Cell-Mediated
Rejection in Kidney Transplant Recipients: A Cross-Sectional Study. PLoS One.

[51] Park J., Lin H. Y., Assaker J. P., Jeong S., Huang C. H., Kurdi T., Lee K., Fraser K., Min C., Eskandari S., Routray S., Tannous B., Abdi R., Riella L., Chandraker A., Castro C. M., Weissleder R., Lee H., Azzi J. R. (2017). Integrated Kidney Exosome Analysis for the Detection
of Kidney Transplant Rejection. ACS Nano.

[52] Schweke H., Pacesa M., Levin T., Goverde C. A., Kumar P., Duhoo Y., Dornfeld L. J., Dubreuil B., Georgeon S., Ovchinnikov S., Woolfson D. N., Correia B. E., Dey S., Levy E. D. (2024). An Atlas
of Protein Homo-Oligomerization Across Domains
of Life. Cell.

[53] Gotte G., Menegazzi M. (2023). Protein Oligomerization. Int.
J. Mol. Sci..

[54] Gell D. A., Grant R. P., Mackay J. P. (2012). The Detection
and Quantitation of
Protein Oligomerization. Adv. Exp. Med. Biol..

[55] Lo C. H. (2021). Recent
Advances in Cellular Biosensor Technology to Investigate Tau Oligomerization. Bioeng. Transl. Med..

[56] Parekh P., Martin J., Chen Y., Colon D., Wang H., Tan W. (2008). Using Aptamers to Study Protein–Protein Interactions. Adv. Biochem. Eng. Biotechnol..

[57] Lei Y., Fei X., Ding Y., Zhang J., Zhang G., Dong L., Song J., Zhuo Y., Xue W., Zhang P., Yang C. (2023). Simultaneous Subset Tracing and miRNA Profiling of Tumor-Derived
Exosomes via Dual-Surface-Protein Orthogonal Barcoding. Sci. Adv..

[58] Wang Z., Zhang Y., Wu L., Chen J., Xie S., He J., Zhang Q., Chen H., Chen F., Liu Y., Zhang Y., Zhuo Y., Wen N., Qiu L., Tan W. (2023). An Aptamer-Functionalized DNA Circuit to Establish an Artificial
Interaction between T Cells and Cancer Cells. Angew. Chem. Int. Ed..

[59] Liu S., Li S., Lin J., Li J., Yang H. (2022). Aptamer-Induced-Dimerization
Strategy Attenuates AβO Toxicity through Modulating the Trophic
Activity of PrPC Signaling. J. Am. Chem. Soc..

[60] Liang H., Chen S., Li P., Wang L., Li J., Li J., Yang H.-H., Tan W. (2018). Nongenetic Approach for Imaging Protein
Dimerization by Aptamer Recognition and Proximity-Induced DNA Assembly. J. Am. Chem. Soc..

[61] Degn S. E., Tolar P. (2025). Towards a Unifying Model for B-Cell Receptor Triggering. Nat. Rev. Immunol..

[62] Chen J., Qiu S., Li W., Wang K., Zhang Y., Yang H., Liu B., Li G., Li L., Chen M., Lan J., Niu J., He P., Cheng L., Fan G., Liu X., Song X., Xu C., Wu H., Wang H. (2023). Tuning Charge
Density of Chimeric Antigen Receptor Optimizes Tonic Signaling and
CAR-T Cell Fitness. Cell Res..

[63] Petrova Z. O., Han L., Tsutsui Y., Sheetz J. B., Ashtekar K. D., Lemmon M. A. (2026). The Role
of Kinase Domain Dimerization in EGFR Activation. Structure.

[64] Liu J., Tang H., Xu C., Zhou S., Zhu X., Li Y., Prézeau L., Xu T., Pin J.-P., Rondard P., Ji W., Liu J. (2022). Biased Signaling
Due
to Oligomerization of the G Protein-Coupled Platelet-Activating Factor
Receptor. Nat. Commun..

