# Supplementary material for: Discriminating Post-Transplant Rejection from Infection by Detecting TCR-CD3 Oligomerization on Extracellular Vesicles Using a Ratiometric Caliper Probe
Source: J Am Chem Soc. 2026 May 28;148(22):23357–71. doi: 10.1021/jacs.6c07971 (PMC13266703; doi:10.1021/jacs.6c07971)
Supplement: Supplementary file 1 [file ja6c07971_si_001.pdf]

Supporting Information

Discriminating Post-Transplant Rejection from Infection by Detecting TCR–CD3 Oligomerization on Extracellular Vesicles Using a Ratiometric Caliper Probe

Wen Yin#, Haitian Chen#, Shu Xiao, Jun Zheng, Xuegang Zhao, Linda Fan, Xing Lv, Haijin Lv, Qing Yang, Jia Yao, Xiaofeng Yuan*, Yang Yang*, and Mo Yang*

[#] These authors contributed equally: Wen Yin, Haitian Chen.

Corresponding Author

* yuanxf5@mail.sysu.edu.cn

* yysysu@163.com

* mo.yang@polyu.edu.hk

Materials and Methods

Materials and reagents

The used oligonucleotides were custom-synthesized and HPLC-purified by Sangon Biotechnology Co. Ltd. (Shanghai, China), and all the sequences are listed in **Table S5**. Non-fat powdered milk, bovine serum albumin (BSA), and 30% Acryl/Bis solution (29:1), 4SGelred were purchased from Sangon Biotechnology Co. Ltd. (Shanghai, China). N,N,N',N'-tetramethylethane-1,2-diamine (TEMED), ammonium persulfate (APS), and lipopolysaccharide (LPS) were purchased from Sigma-Aldrich (St. Louis, USA). Roswell Park Memorial Institute (RPMI)-1640 cell culture medium, Dulbecco's modified Eagles medium (DMEM) cell culture medium, fetal bovine serum (FBS), EV-depleted FBS, and phosphate-buffered saline (PBS, 0.01 M, pH 7.4) were obtained from Gibco, Life Technologies Co. (Grand Island, NY, USA). RIPA lysis buffer, phenylmethanesul-fonyl fluoride (PMSF), Proteinase K, tris buffered saline with Tween-20 (TBST), BeyoECL Star Kit, DAB Horseradish Peroxidase Color Development Kit, and BCA Protein Assay Kit, BeyoGel Blue Native PAGE Sample Buffer were purchased from Beyotime Biotechnology Co. (Shanghai, China). Granulocyte-macrophage colony-stimulating factor (GM-CSF), interleukin-4 (IL-4), interferon-gamma (IFN-γ), carboxyfluorescein succinimidyl ester (CFSE), and mouse CD8+ T cell isolation kit were purchased from Yeasen Biotech Co., Ltd (Shanghai, China). Brij-96 was purchased from Aladdin (Shanghai). All the chemicals used were of analytical reagent grade, and they were directly used without additional purification. All solutions were prepared with Milli-Q water (resistivity of 18.2 MΩ·cm) or PBS buffer.

Cell lines and cell culture

Human T cell leukemia cell line (Jurkat) and human breast cancer epithelial cell line (MDA-MB-231) were obtained from Sangon Biotechnology Co. Ltd. (Shanghai, China). Jurkat cells were cultured in RPMI-1640 medium supplemented with 10% FBS. MDA-MB-231 cells were cultured in DMEM medium supplemented with 10% FBS. All cells

were cultured at 37 °C in a humidified 5% CO_2 incubator (Thermo Fisher Scientific, USA). Jurkat cells grow in suspension with a concentration between 2×10^5 and 1×10^6 cells mL^{-1}.

Model EVs isolation from cell culture medium

Model EVs were obtained from cell culture medium by differential ultracentrifugation. Once 5×10^5 cells mL^{-1} of density was achieved, the culture medium was replaced with RPMI-1640 medium supplemented with 1% EV-depeleted FBS. After 48 h of EV secretion, the cell medium was collected and centrifuged at 300 *g* for 10 min and 3,000 *g* for 30 min to remove cells and cell debris. The supernatant was further centrifuged at 10,000 *g* for 30 min to remove large granular vesicles and protein aggregates. The supernatant was then transferred to ultracentrifuge tubes for ultracentrifugation at 100,000 *g*, 4 °C for 70 min using a SW 32 Ti rotor (Beckman Coulter, USA) and operated using Optima XPN-100 (Beckman, USA). Following that, the EVs were washed with PBS buffer, ultracentrifuged at 100,000 *g*, 4 °C for 70 min, re-suspended in 200 μL of PBS, and stored at −80 °C until use.

Characterization of Jurkat EVs by TEM and nano-flow cytometry

A droplet of Jurkat EVs was deposited on an ultra-thin carbon film copper mesh and dried at room temperature for 4 h. Then the sample on copper was washed by ddH_2O for five times and dried at room temperature for 4 h. The morphologies of EVs were observed on a TEM (JEM-2100F, JEOL, Japan). To characteristic the size distribution and CD3 expression of Jurkat EVs, the isolated EVs were incubated with FITC anti-CD3 antibody (317305, Biolegend, San Diego, CA, USA) at 37 ºC for 30 min in the dark. Nano-flow cytometry was performed using NanoFCM (Fuliu, China).

Western blot analysis

Jurkat EVs and cells were lysed in RIPA buffer with 1% PMSF, and the protein concentration was measured using a BCA Protein Assay Kit for protein preparations. Equivalent micrograms of proteins were loaded for each sample onto 12% SDS-PAGE.

Following electrophoresis at 100 V, the proteins were transferred from gel onto a PVDF membrane (0.22 μm, Millipore). The membrane was blocked with 5% non-fat milk in 1 × TBST overnight at 4 ºC and incubated with the following primary antibodies overnight at 4 ºC: anti-CD63 rabbit monoclonal antibody (ab134045, Abcam, Cambridge, UK), anti-CD9 rabbit monoclonal antibody (ab263019, Abcam, Cambridge, UK), and anti-CD3 rabbit monoclonal antibody (85061, Cell Signaling Technology, Beverly, MA, USA). Following incubation with HRP-conjugated secondary antibody (ab6721, Abcam, Cambridge, UK) for 1 h at room temperature, the immunoreactive bands were developed with BeyoECL Star Kit and visualized by an imaging system (ChemiDoc, Bio-Rad, USA).

Binding affinity determination

Binding affinities of aptamer switch probes (TA10, TA14, TA18, TA20) against Jurkat EVs were determined by incubating a range of probe concentrations (0 nM – 250 nM) with 1 × 10^{10} particles mL^{-1} of Jurkat EVs at 37 ºC for 2 h. The total volume of the reaction system was 50 μL and was carried out in a 96-well microplate (3694, Corning, USA). Binding events were analyzed by a microplate reader (SpectraMax i3X, Molecular Devices, USA) with the fluorescence excitation at 485 nm and emission at 520 nm. The equilibrium dissociation constant (K_d) values were calculated based on the following equation:

$$Y = B_{max} \times \frac{X}{(K_d + X)}$$

where Y is the fluorescence intensity, X is the probe concentration, and B_{max} is the maximum number of binding sites.

Hybridization of the aptamer probes

To form caliper probes (e.g., Caliper-17), a mixture of 1 μL of 10 μM A10-T1* and 1 μL of 10 μM A20-T2* were added into 1 μL of 10 μM T1-1-T2 and incubated at 37 ºC for 2 h. To confirm the formation of the aptamer probes, oligos were carried out in 8% native polyacrylamide gel electrophoresis (PAGE) in 0.5 × TBE, performed at 90 V on an electrophoresis apparatus (PowerPac Basic, Bio-Rad, USA), stained by 4SGelred, and imaged on a gel imaging system (ChemiDoc, Bio-Rad, USA).

Fluorescence kinetics analysis of caliper probe binding to EVs

To assess the fluorescence kinetics of caliper probe binding to CD3, 200 nM of the probe was mixed with either model EVs (1×10^{10} particles mL^{-1}) or 5 μL of plasma in a 96-well microplate. Each well was adjusted to a final volume of 50 μL with PBS. The fluorescence signals were continuously monitored at 37 °C using a microplate reader. Fluorescence intensity was recorded using the following excitation/emission (Ex/Em) settings: 485/520 nm for FAM fluorescence and 580/605 nm for ROX fluorescence.

Isolation of plasma EVs by ultrafiltration

Plasma sample was centrifuged at 18,000 *g* for 30 min at 4 °C and the supernatant was carefully transferred to a new tube avoiding any pellets. BCA assay was performed to determine protein concentration. Proteinase K solution (150 μg mL^{-1}) was added to 10 mg protein sample and incubated at 37 °C for 30 min, following 60 °C for 10 mins to inactivate Proteinase K. The plasma sample was then added to a 100kDa MWCO ultrafiltration device (Amicon® Ultra, Millipore) and centrifuged at 3,700 *g* for 15 min until the volume is reduced to 50 μL. Add 450 μL PBS onto the filter membrane and repeat the wash step a total of three times. The final 50 μL of plasma EVs was transferred to a new tube, and the ultrafiltration device was inverted and centrifuged at 2,000 *g* for 5 min to collect the retentate. To characterize the size distribution of plasma EVs, 5 μL of EVs suspension was diluted to 1 mL using PBS buffer and analyzed using nanoparticle tracking analysis (NTA, NanoSight NS300, Malvern, UK).

Generation and maturation of bone marrow-derived dendritic cells (BMDCs)

Bone marrow cells were isolated by flushing the femurs and tibias of BALB/c mice. After red blood cell lysis, the cells were seeded into 6-well plates at a density of 1×10^6 cells mL^{-1}. Differentiation into immature BMDCs was induced by culturing the cells in RPMI-1640 complete medium supplemented with 20 ng mL^{-1} GM-CSF and 20 ng mL^{-1} IL-4 for 8 days. Following the differentiation period, the non-adherent and loosely adherent cells in suspension were harvested. To evaluate the purity of the generated BMDCs, a portion of

the collected cells was stained with APC anti-mouse CD11c (117309, Biolegend, San Diego, CA, USA) and analyzed by flow cytometry. For maturation, the harvested cells were re-seeded and stimulated with 1 μg mL^{-1} LPS and 20 ng mL^{-1} IFN-γ for 18 h. To assess the maturation status, the stimulated cells were collected and stained with CoraLite® Plus 405 anti-mouse MHC class II (CL405-65122, Proteintech, Rosemont, IL, USA), PE anti-mouse CD80 (600055, Biolegend, San Diego, CA, USA), and FITC anti-mouse CD86 (159219, Biolegend, San Diego, CA, USA). The expression of these maturation markers was subsequently evaluated using flow cytometry.

Isolation and purification of CD8+ T cells

Spleens were harvested from C57BL/6 mice and mechanically disrupted to obtain single-cell suspensions. Following red blood cell lysis, the splenocytes were resuspended at a density of 1×10^8 cells mL^{-1}. For CD8+ T cell enrichment, the 100 μL of cell suspension was mixed with 2 μL of an antibody cocktail and incubated on ice for 10 min. Subsequently, 20 μL of streptavidin magnetic beads were added to the mixture, followed by an additional 10-min incubation on ice. The sample was then diluted with 2.5 mL of separation buffer and placed on a magnetic rack for 5 min. The supernatant, containing the purified and untouched CD8+ T cells, was carefully collected. To verify the isolation efficiency, the enriched cells were stained with APC anti-mouse CD3 (100235, Biolegend, San Diego, CA, USA) and PE anti-mouse CD8α (553032, BD Biosciences, San Jose, CA, USA), and the purity was assessed by flow cytometry. For subsequent proliferation assays, the purified CD8+ T cells were pre-labeled with CFSE prior to co-culture assay.

Mixed lymphocyte reaction (MLR) assay

To establish the MLR model, mature BMDCs and purified CD8+ T cells (pre-labeled with CFSE for proliferation tracking) were seeded into 24-well plates at a ratio of 1:5. The co-culture was maintained in RPMI-1640 medium supplemented with 1% EV-depleted FBS and 1% penicillin-streptomycin. The plates were incubated at 37°C in a humidified atmosphere containing 5% CO_2. After 3 days of co-culture, the cells were harvested to

evaluate T cell activation and proliferation. The collected cells were stained with PE/Cyanine7 anti-mouse CD3 (100219, Biolegend, San Diego, CA, USA) and APC anti-mouse CD25 (102011, Biolegend, San Diego, CA, USA), and subsequently analyzed by flow cytometry. After 5 days of co-culture, the cell culture supernatants were collected, and the secretion of interferon-gamma (IFN-γ) was quantified using an enzyme-linked immunosorbent assay (ELISA) (Servicebio, Cat# GEM0006-48T) according to the manufacturer's instructions. To isolate EVs, the cell culture supernatants were collected after 24 h of co-culture. To remove intact cells, dead cells, and cellular debris, the collected supernatants were subjected to sequential differential centrifugation at 500 g for 5 min, 2,000 g for 15 min, and 10,000 g for 30 min. The pre-cleared supernatants were then transferred to 100 kDa MWCO ultrafiltration tubes and centrifuged at 3,700 g for 15 min to concentrate the EVs. Following concentration, the retained EVs were washed three times with PBS using the same ultrafiltration conditions. Finally, the purified EVs were resuspended in 50 μL of PBS for downstream applications.

Blue Native PAGE (BN-PAGE) analysis of TCR-CD3 oligomerization

To evaluate the oligomeric state of the TCR-CD3 complex, cells and EVs collected from the MLR assay were lysed in a native lysis buffer supplemented with 0.5% Brij-96 on ice for 30 min. The lysates were then centrifuged at 15,000 g for 15 min at 4°C to remove insoluble cellular debris. The resulting supernatants were collected and mixed with 2× BeyoGel Blue Native PAGE Sample Buffer. The protein complexes were subsequently loaded onto a 3.5 – 12% gradient polyacrylamide gel and resolved by electrophoresis at a constant voltage of 100 V for 2 h. Following separation, the proteins were transferred onto a PVDF membrane (0.45 μm, Millipore) for 90 min. The membrane was blocked with 5% non-fat milk in TBST for 1 h at room temperature. For immunodetection, the membrane was incubated overnight at 4 °C with an anti-CD3ε primary antibody (78588T, Cell Signaling Technology, Beverly, MA, USA). After washing three times with TBST, the membrane was incubated with HRP-conjugated secondary antibody (ab6721, Abcam,

Cambridge, UK) for 1 h at room temperature. Following three final washes with TBST, the protein bands were visualized using an enhanced ECL detection system.

Nanoflow-cytometry characterization of EVs

To characterize the surface markers of the EVs isolated from the MLR system, 20 μL of the purified EV suspension was mixed with 1 μL of a prepared antibody cocktail. The antibody cocktail consisted of 0.1 μL FITC anti-mouse CD3ε (100203, Biolegend, San Diego, CA, USA), 0.1 μL PE anti-mouse CD8α (553032, BD Biosciences, San Jose, CA, USA), 0.1 μL APC anti-mouse TCRβ (109211, Biolegend, San Diego, CA, USA), and 0.7 μL of PBS. The mixture was gently vortexed and incubated at 37°C for 30 min in the dark. Following incubation, nano-flow cytometry analysis was performed to evaluate the expression of these surface proteins using a NanoFCM instrument (Fuliu, China).

Skin transplantation mouse model

Eight-week-old male C57BL/6 mice and BALB/c mice were obtained from the Guangdong Yaokang Biotechnology Co., Ltd. The mice were fed in a specific pathogen-free (SPF) environment with a 12-h light/12-h dark cycle, a temperature of 20 – 24 °C, and a relative humidity of 50 – 60%. The use of laboratory animals and protocol for mouse skin transplantation experiments were approved by the Institutional Animal Care and Use Committee of Jennio Biotech Co., Ltd, China, approval no. JENNIO-IACUC-2025-A018.

To perform skin transplantation, mice were anesthetized with isoflurane (Rayward Life Technology Co., Ltd, Shenzhen, China), shaved, and disinfected with 10% povidone iodine. A patch of back skin (each approximately ~ 1 cm^2) was removed from the donor BALB/c or C57BL/6 mice and kept in sterile saline on ice until the grafting procedure. In recipient C57BL/6 mice, the skin on the back was removed using surgical scissors to create a defect of similar size to the donor skin. The donor skin was then placed over the site to cover the wound defect. The site was sutured and then protected by covering a commercial bandage and firmly wrapped around the body of the animal. Monitor daily for signs of rejection.

CLP-induced sepsis mouse model

Eight-week-old male C57BL/6 mice were obtained from the Guangdong Yaokang Biotechnology Co., Ltd. The use of laboratory animals and protocol for mouse CLP experiments were approved by the Institutional Animal Care and Use Committee of Jennio Biotech Co., Ltd, China, approval no. JENNIO-IACUC-2025-A018.

Briefly, mice were anesthetized with isoflurane and made a midline abdominal incision. The cecum was mobilized, ligated at the half the distance between distal pole and the base of cecum (mid-grade sepsis), and punctured once with an 18G needle to induce polymicrobial peritonitis. The cecum was relocated into the abdominal cavity without spreading feces from the cecum onto the abdominal wall wound margins. The peritoneum, fasciae and abdominal musculature were closed by applying running sutures. After surgery, 1 mL of physiological saline solution was subcutaneously injected into the mice for fluid resuscitation. All mice had unlimited access to food and water both pre-and postoperatively. In the Rej-Ing group, C57BL/6 mice first underwent a skin transplant procedure. Once complete graft necrosis was observed, the CLP model was subsequently performed as described above.

H&E and IHC staining

The mice were euthanized, and the skin grafts were harvested and fixed with 4% paraformaldehyde, followed by dehydration in an ethanol gradient. After paraffin embedding, the samples were sectioned into 5 μm thickness slices and processed for H&E staining. For CD3 IHC staining, the skin sections were subjected to deparaffinization, rehydration, and heat-induced epitope retrieval and were subsequently blocked with 5% BSA solution for 30 min at room temperature. Afterward, samples were incubated with anti-CD3 antibody (ab56313, Abcam, Cambridge, UK) overnight at 4 ^{o}C, and then they were incubated with an HRP-conjugated secondary antibody for 1 h at room temperature. After washing with PBS, CD3 positive T cells were detected using DAB Kit and observed by a panoramic scanning microscope (TissudFAXS SL Spectra, TissueGnostics, Austria).

Cytokine measurements

The peripheral blood of mice was collected in EDTA-coated tubes following the procedure for blood collection from the submandibular vein. After centrifuged at 3,000 *g* for 15 min at 4 °C, the plasma of supernatant was transferred to new tubes. TNF-α, IL-IL-6, and MCP-1 concentrations in plasma samples were tested by the ELISA kits (88-7324, 88-7064, 88-7391, Invitrogen, Thermo Fisher Scientific Inc., USA).

Flow cytometry analysis of mouse peripheral blood T cells

Peripheral blood (50 μL) was collected from mice via the submandibular vein into EDTA-coated microtubes. The blood was diluted 1:1 with PBS and incubated with RBC lysis buffer at room temperature for 5 – 10 min to lyse red blood cells. Samples were centrifuged at 400 *g* for 5 min and washed twice with PBS containing 1% BSA. The cell pellet was resuspended in 100 μL PBS and stained with fluorochrome-conjugated surface antibodies against CD3, CD4, and CD8 (Biolegend, San Diego, CA, USA) for 30 min at 4 °C in the dark. After surface staining, cells were washed once with PBS and then fixed using 100 μL of fixation buffer. Samples were incubated at room temperature for 20 min. Following fixation, cells were washed and resuspended in permeabilization buffer (Invitrogen, Thermo Fisher Scientific Inc., USA) and incubated with Brilliant Violet 421™ anti-GzmB antibody (396413, Biolegend, San Diego, CA, USA) for 30 min at 4 °C in the dark. After intracellular staining, cells were washed twice with permeabilization buffer, then once with PBS, and detected by flow cytometry.

RNA sequencing of mouse spleen tissues

Mouse spleens were harvested under sterile conditions, immediately snap-frozen in liquid nitrogen, and stored at −80 °C until processing. Total RNA was extracted using the TRIzol reagent (Invitrogen) following the manufacturer’s protocol. RNA purity and concentration were assessed using a NanoDrop spectrophotometer (Thermo Scientific), and RNA integrity was evaluated using an Agilent 2100 Bioanalyzer (Agilent Technologies). Only high-quality RNA samples with RNA Integrity Number (RIN) ≥ 8.0 were used for library

preparation. Messenger RNA (mRNA) was enriched using poly-T oligo-attached magnetic beads, fragmented, and reverse transcribed into complementary DNA (cDNA). Following end repair, A-tailing, adapter ligation, and PCR amplification, the libraries were purified and validated for quality and fragment size distribution using the Bioanalyzer 2100 system. The sequencing was performed on an Illumina NovaSeq 6000 platform, generating paired-end 150 bp reads. Raw sequencing data were subjected to quality control using FastQC, and adapters and low-quality reads were removed using Trim Galore. Clean reads were aligned to the mouse reference genome GRCm39 (Ensembl release 113) using HISAT2. Based on the alignment results, transcript assembly and reconstruction were performed using StringTie. Gene expression levels were then quantified for each sample using RSEM and normalized as Fragments Per Kilobase of transcript per Million mapped reads (FPKM) to account for transcript length and sequencing depth. The RNA libraries were sequenced on the Illumina sequencing platform by Guanzhou Xiangyan Biotechnology Co.,Ltd.

Collection of clinical samples

The study was conducted in accordance with the ethical approval of the Departmental Research Committee of Hong Kong Polytechnic University (Reference Number: HSEARS20230821001). Blood samples were collected from rejection patients (n = 17), infection patients (n = 10), and concurrent rejection and infection (Rej-Inf, n = 7), as determined by tissue biopsy and microbial identification. The plasma was isolated by collecting blood samples in EDTA tubes followed by centrifugation at 3,000 g for 15 min. Plasma samples were stored at −80 °C until analysis. The entire analytical processes, including plasma sample processing and the Caliper-17 assay, were performed by operators who were completely blinded to the patients' clinical diagnoses, biopsy results, and microbiological culture data. The fluorescence data were recorded independently as "unknown samples." Only after all the fluorescence ratios were calculated were the results unblinded and matched with the corresponding clinical adjudications for the final statistical analysis.

Table. S1 Binding ability of the caliper probe with different permutation and combination

	TA10-TA20		TA10-TA10		TA20-TA10	
	ROX	FAM	ROX	FAM	ROX	FAM
K_d (nM)	35.20	61.70	35.20	35.20	61.70	35.20
Base number of A	49.00	59.00	49.00	49.00	59.00	49.00
R (nm)	16.17	19.47	16.17	16.17	19.47	16.17
Area (nm^2)	615.76	297.58	615.76	205.25	892.74	205.25
[a]Binding abililty	17.49	4.82	17.49	5.83	14.47	5.83

[a]Binding ability was defined as Area / K_d

Table S2. Participant demographic and clinical characteristics.

**Characteristics**			
Group	Rejection	Infection	Rej-Inf
Total	17	10	7
**Gender**			
Female, No. (%)	6 (35)	2 (20)	1 (14)
Male, No. (%)	11 (65)	8 (80)	6 (86)
**Age (mean ± s.d.)**			
	40 ± 25	60 ± 8	46 ± 17
**TCMR grading**			
Mild	9	\	5
Moderate	7	\	2
Severe	2	\	\
**Microbiological identification**			
EB Virus	\	2	1
Bacteria	\	4	4
Fungi	\	4	2
**Immunosuppressant level**			
Tacrolimus, No. (ng/mL)	8 (7.3 ± 4.7）	3 (5.5 ± 2.3）	3 (7.8 ± 1.7）
Cyclosporine A, No. (ng/mL)	5 (168.3 ± 65.9）	4 (97.1 ± 39.5）	2 (142.6 ± 58.2）
Sirolimus, No. (ng/mL)	4 (10.0 ± 2.5）	3 (5.9 ± 2.5）	2 (7.9 ± 3.8）

Table S3. Confusion matrix at the cut-off of 0.69.

	Predicted: Rejection ($\geqslant$ 0.69)	Predicted: Infection (< 0.69)
Actual: Rejection present (*n* = 24, 17 Rejection + 7 Rej-Inf)	True positive (TP) = 17	False negative (FN) = 7
Actual: Infection only (*n* = 10, 10 Infection)	False positive (FP) = 1	True negative (TN) = 9

Note: The overall accuracy is calculated as the proportion of true results (both true positives and true negatives) among the total number of cases examined.

$$\text{Accuracy} = \frac{\text{TP+TN}}{\text{TP+FP+TN+FN}} = \frac{17+9}{34} = 76.47\% \approx 76\%.$$

Table S4. Intra- and inter-assay reproducibility of clinical samples by Caliper-17.

Group	Clinical samples	Intra-assay (n = 3)			Inter-assay (n = 3)		
		Mean	s.d.	CV (%)	Mean	s.d.	CV (%)
Rejection	R1	1.04	0.13	12.10	1.03	0.12	12.00
	R2	0.63	0.02	2.56	0.64	0.08	11.82
	R3	0.60	0.03	4.62	0.60	0.04	7.34
Infection	I1	0.44	0.01	2.98	0.43	0.02	5.50
	I2	0.40	0.02	4.84	0.40	0.03	7.39
	I3	0.12	0.01	5.28	0.11	0.01	13.06
Rej-Inf	R-I1	0.66	0.02	3.68	0.69	0.05	6.78
	R-I2	0.77	0.01	1.33	0.77	0.09	11.44
	R-I3	1.07	0.01	1.06	1.08	0.07	6.82

Table. S5 Sequences of the used oligonucleotides

**Name**	**Sequences (5' – 3')**
T1	AGTAACTCAAGCCATGCACCATAG(**BHQ1)**
A9-T1*	(**FAM**)GCCGCGGGGTGGGTCTAGTGTGGATGTTTAGGGGGCGGCC CCCGCGGCCTATGGTGCATGGCTTGAGTTACT
A10-T1*	(**FAM**)GCCGCGGGGTGGGTCTAGTGTGGATGTTTAGGGGGCGGCA CCCCGCGGCCTATGGTGCATGGCTTGAGTTACT
A14-T1*	(**FAM**)GCCGCGGGGTGGGTCTAGTGTGGATGTTTAGGGGGCGGCA CCCACCCCGCGGCCTATGGTGCATGGCTTGAGTTACT
A18-T1*	(**FAM**)GCCGCGGGGTGGGTCTAGTGTGGATGTTTAGGGGGCGGCC TAGACCCACCCCGCGGCCTATGGTGCATGGCTTGAGTTACT
A20-T1*	(**FAM**)GCCGCGGGGTGGGTCTAGTGTGGATGTTTAGGGGGCGGCC ACTAGACCCACCCCGCGGCCTATGGTGCATGGCTTGAGTTACT
T2	ACGTGTTCGAGTACTAGTTAGATA(**BHQ2**)
A10-T2*	(**ROX**)GCCGCGGGGTGGGTCTAGTGTGGATGTTTAGGGGGCGGCA CCCCGCGGCTATCTAACTAGTACTCGAACACGT
A20-T2*	(**ROX**)GCCGCGGGGTGGGTCTAGTGTGGATGTTTAGGGGGCGGCC ACTAGACCCACCCCGCGGCTATCTAACTAGTACTCGAACACGT
A20-T3*	(**ROX**)GCCGCGGGGTGGGTCTAGTGTGGATGTTTAGGGGGCGGCC ACTAGACCCACCCCGCGGCTATCTAACTAGTACT
T1-1-T3	AGTAACTCAAGCCATGCACCATAG(**iBHQ1dT**)AGTACTAGTTAGAT A(**BHQ2**)
T1-1-T2	AGTAACTCAAGCCATGCACCATAG(**iBHQ1dT**)ACGTGTTCGAGTAC TAGTTAGATA(**BHQ2**)
T1-10-T2	AGTAACTCAAGCCATGCACCATAG(**iBHQ1dT**)TTTTTTTTTACGTGT TCGAGTACTAGTTAGATA(**BHQ2**)
T1-20-T2	AGTAACTCAAGCCATGCACCATAG(**iBHQ1dT**)TTTTTTTTTTTTTTT

	TTTTACGTGTTCGAGTACTAGTTAGATA(**BHQ2**)
OSJ-T3	(**FAM**)GCCGCGGGGTGGGTCTAGTGTGGATGTTTAGGGGGCGGC
Random sequence (RS)	(**FAM**)ACTACTCTTCTCCGAGCCGGTCGAATCTATAGCTTAC

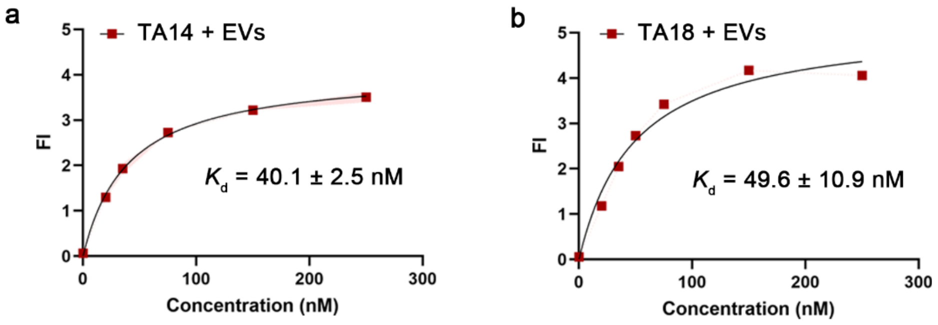

Figure S1. Determination of the dissociation constants (K_d) for TA14 (a) and TA18 (b) via fluorescence saturation curves.

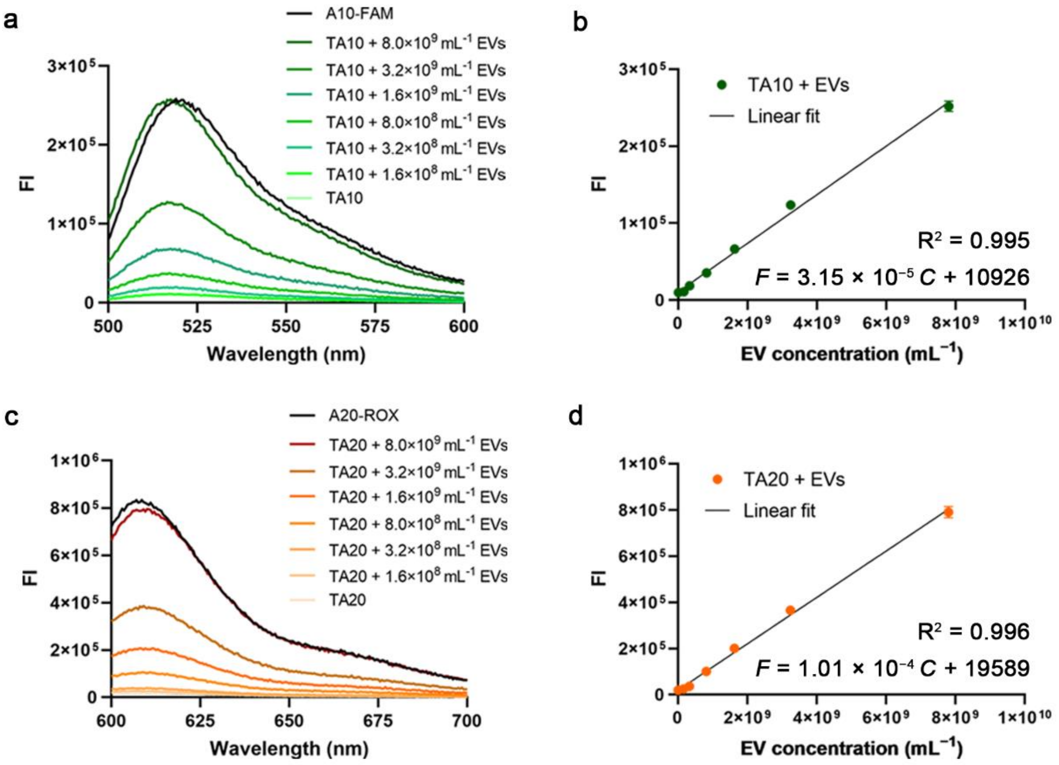

Figure S2. EV concentration-dependent fluorescence response and corresponding linear regression curves for 200 nM TA10 (a,b) and TA20 (c,d).

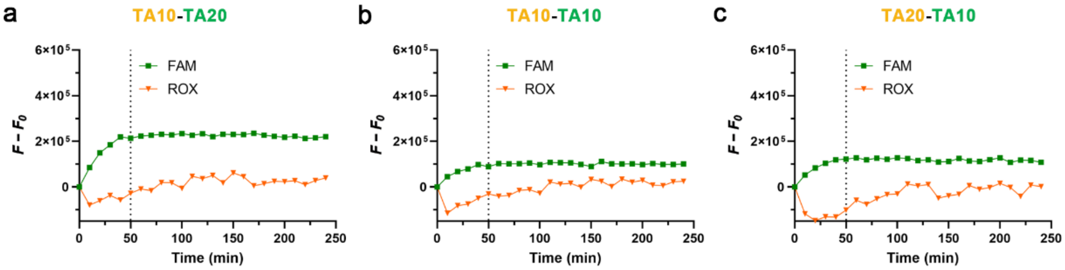

Figure S3. Fluorescence kinetic curve of TA10-TA20 (a), TA10-TA10 (b), and TA20-TA10 (c) in the absence of EVs.

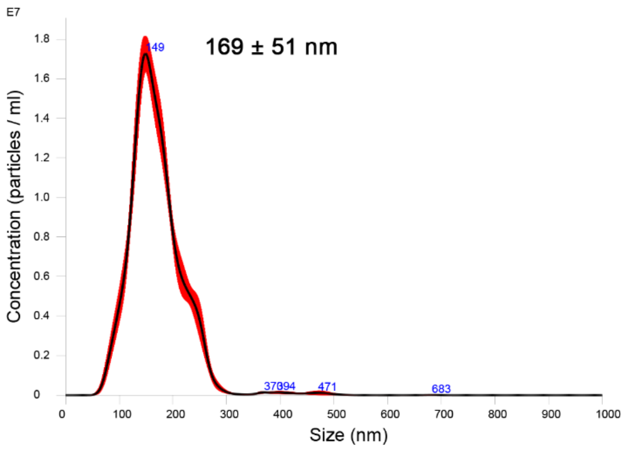

Figure S4. Size distribution of plasma EVs measured by NTA.

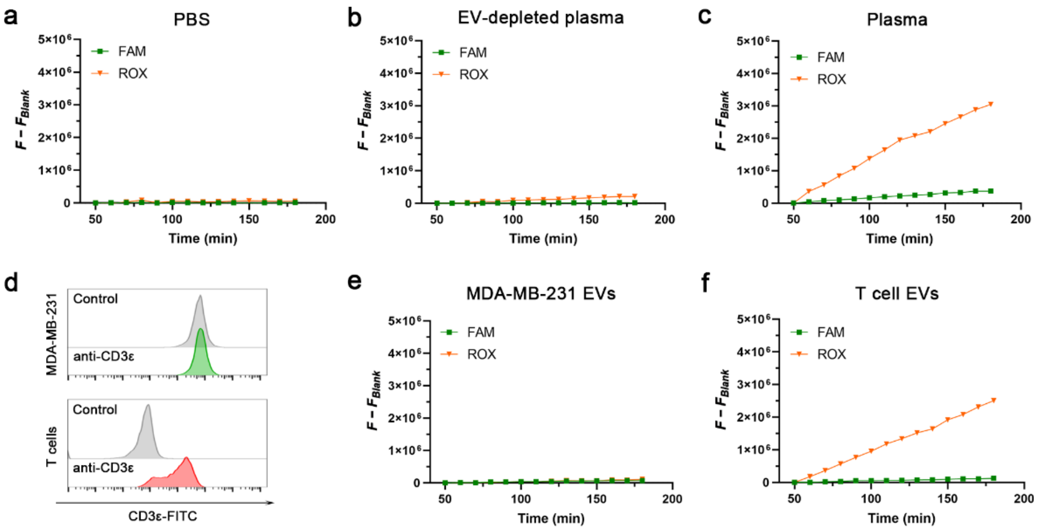

Figure S5. Fluorescence kinetic curves of the caliper probe interacting with (a) PBS, (b) EV-depleted plasma, (c) plasma, (e) MDA-MB-231 EVs, and (f) T cell EVs. (d) CD3 expression of MDA-MB-231 cells and T cells was detected by flow cytometry via anti-CD3ε-FITC staining.

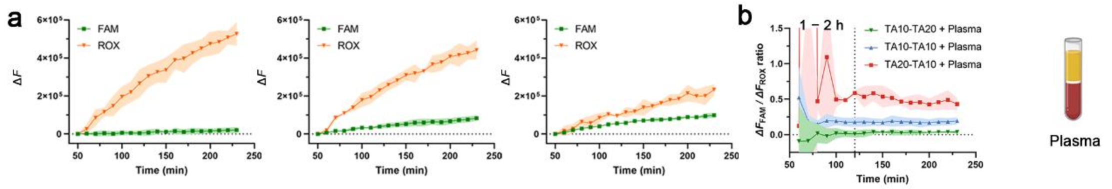

Figure S6. (a) Fluorescence kinetic curves of caliper probes interacting with mouse plasma (mean ± s.d., $n = 3$). (b) The ΔF_{FAM} / ΔF_{ROX} ratio reflects the relative fluorescence response of the caliper probes upon interaction with mouse plasma.

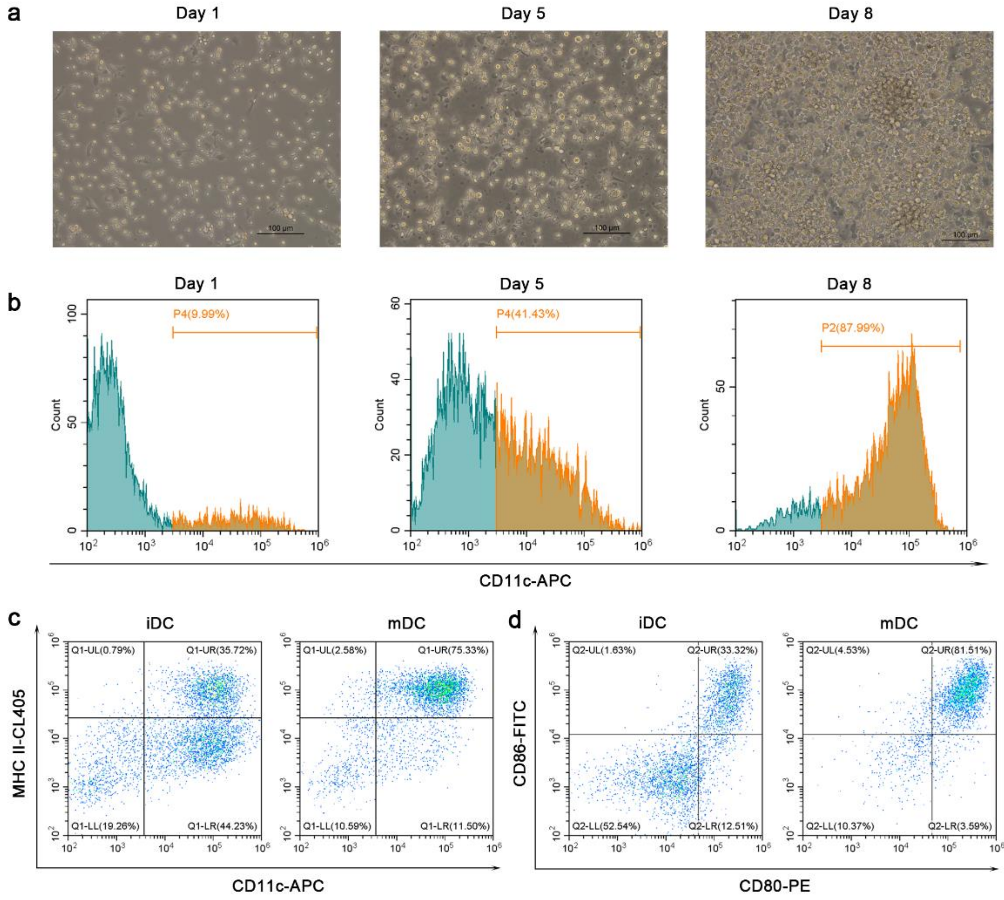

Figure S7. Induced differentiation and maturation of BMDCs from Balb/c mice. (a) Cell morphology and colony formation on days 1, 5, and 8 of differentiation induced by GM-CSF and IL-4. (b) Flow cytometric analysis of CD11c expression percentages on days 1, 5, and 8 of differentiation. (c) Expression levels of MHC II, CD80, and CD86 on BMDCs before and after stimulation with LPS and IFN-γ.

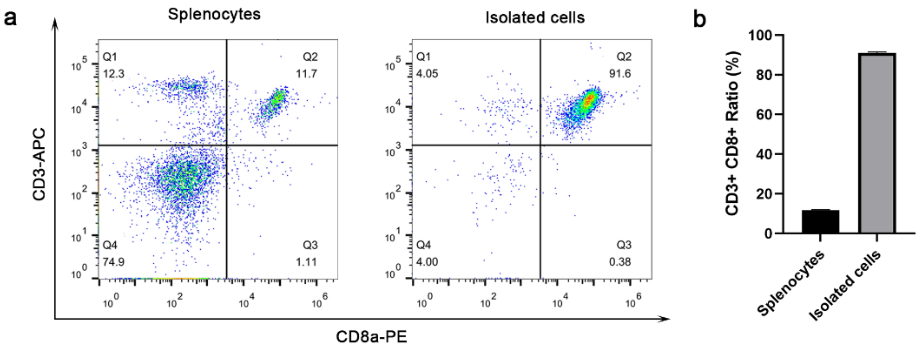

Figure S8. Purity assessment of CD8+ T cells isolated from C57BL/6 mouse spleens. (a) Flow cytometric analysis of CD3 and CD8a expression in total splenocytes and magnetically isolated cells. (b) Quantitative analysis of the corresponding cell population percentages.

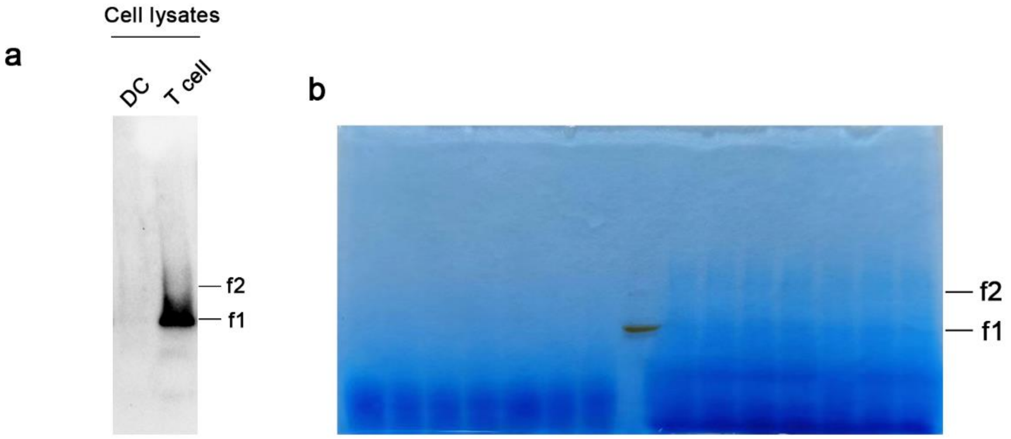

Figure S9. Evaluation of TCR–CD3 protein oligomerization in cell lysates from the DC and T cell by BN-PAGE and anti-CD3ε immunoblotting. The marker protein is ferritin (f1, 440 and f2, 880 kDa forms).

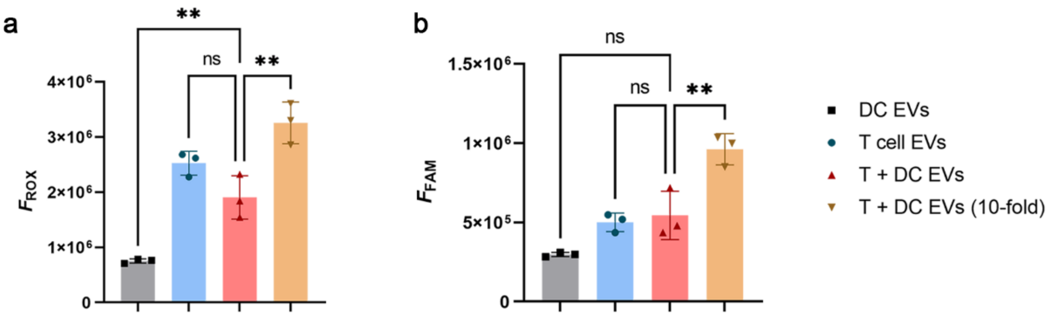

Figure S10. ROX (a) and FAM (b) fluorescence intensities of EVs from the DC, T cell, T+DC, and 10-fold T+DC groups after a 2-h incubation with Caliper-17 (n = 3). Data is presented as mean ± SD. The statistical significance was analyzed using one-way ANOVA following Tukey's multiple comparisons test (**$p < 0.01$, ns = no significance).

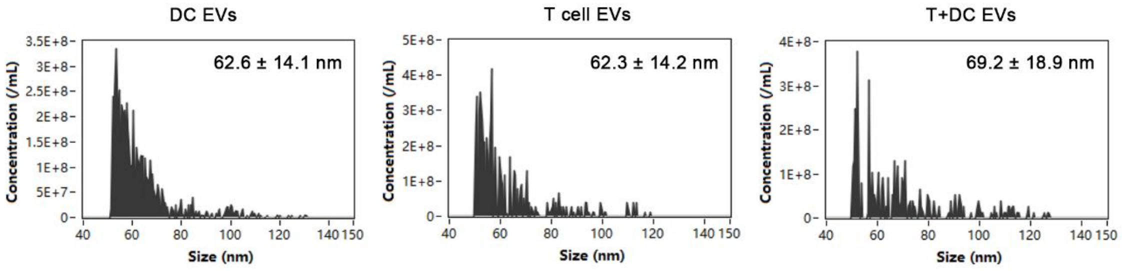

Figure S11. Size distribution of EVs isolated from the supernatant of DC, T cells, and T+DC, measured by nFCM.

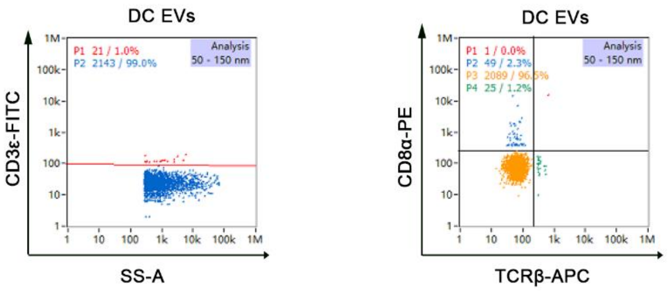

Figure S12. Abundance of CD3ε, CD8α, and TCRβ of DC-derived EVs.

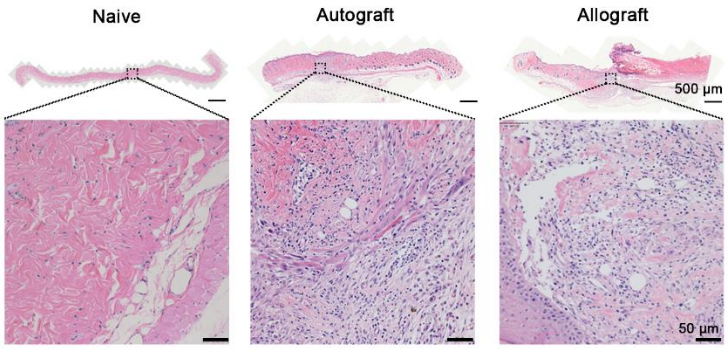

Figure S13. H&E staining of full-thickness skins from naive, autograft, and allograft mice. The junction between the grafted and host skin was marked with a dashed box and enlarged. Scale bar: 50 μm.

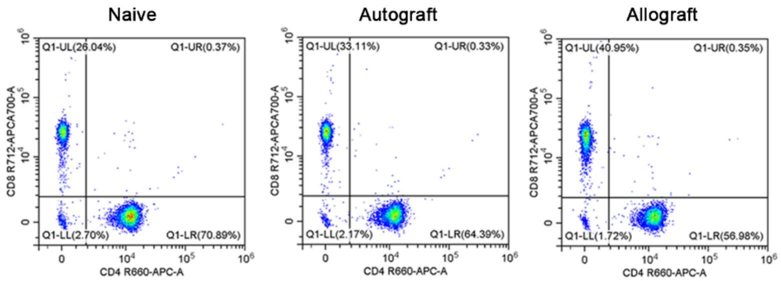

Figure S14. Flow cytometric analysis of CD4 and CD8 expression in peripheral blood T cells from naive, autograft, and allograft mice.

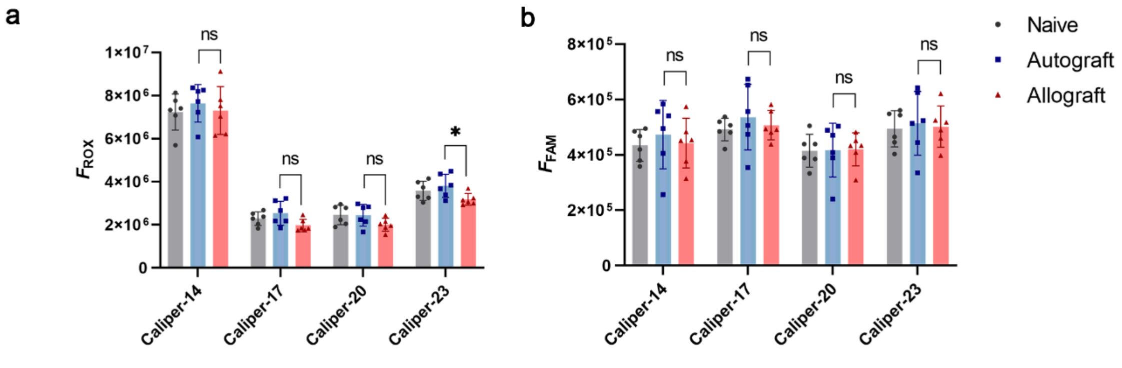

Figure S15. The fluorescence intensities of ROX (a) and FAM (b) in plasma from naive, autograft, and allograft mice were measured using Caliper-14/17/20/23 (mean ± s.d., $n = 6$).

Statistical significance was determined using multiple unpaired two-tailed t-tests. *$p < 0.05$; ns = no significance.

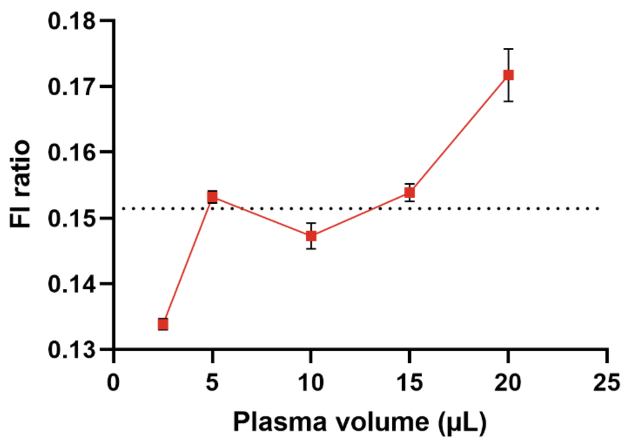

Figure S16. FI ratios measured by Caliper-17 at different plasma input volumes. Dashed line represents the average FI ratio values for the 5, 10, and 15 μL of plasma.

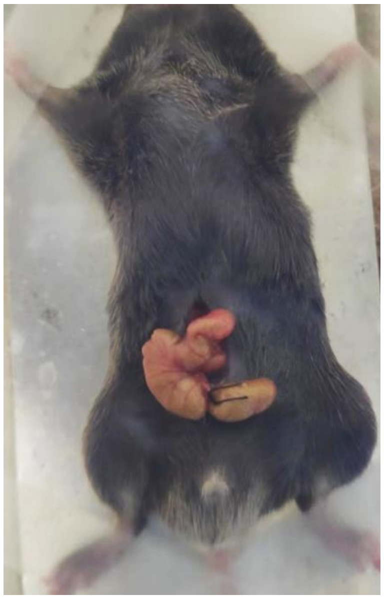

Figure S17. Photo of cecal ligation and puncture (CLP) mouse model. For the induction of mid-grade sepsis resulting in survival rates of ~ 40%, the cecum is ligated at half the distance between distal pole and the base of the cecum.

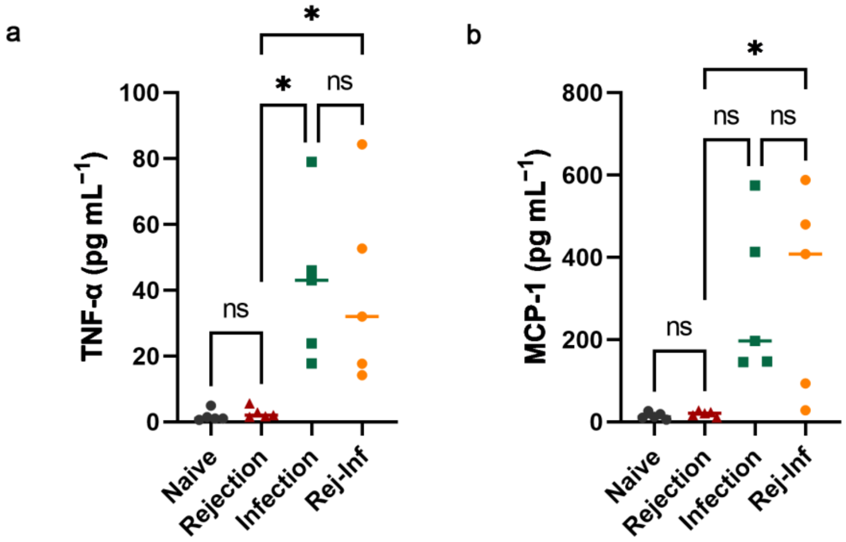

Figure S18. Plasma concentrations of TNF-α (a) and MCP-1 (b) in the Naive, Rejection, Infection, and Rej-Inf groups, measured by ELISA. Statistical significance was assessed using one-way ANOVA followed by Tukey's multiple comparisons test (ns = no significance; $^*p < 0.05$).

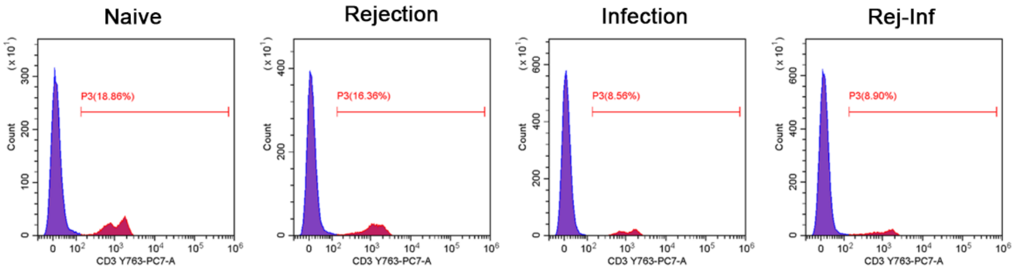

Figure S19. Flow cytometric analysis of the positive ratio of CD3+ T cells in peripheral blood.

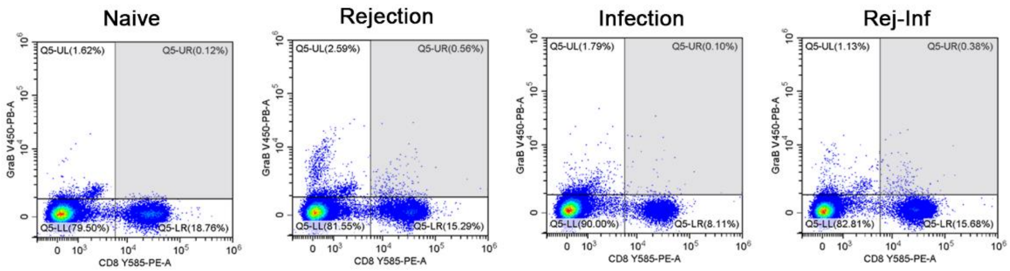

Figure S20. Flow cytometric analysis of the positive ratio of CD8+GzmB+ T cells in peripheral blood.

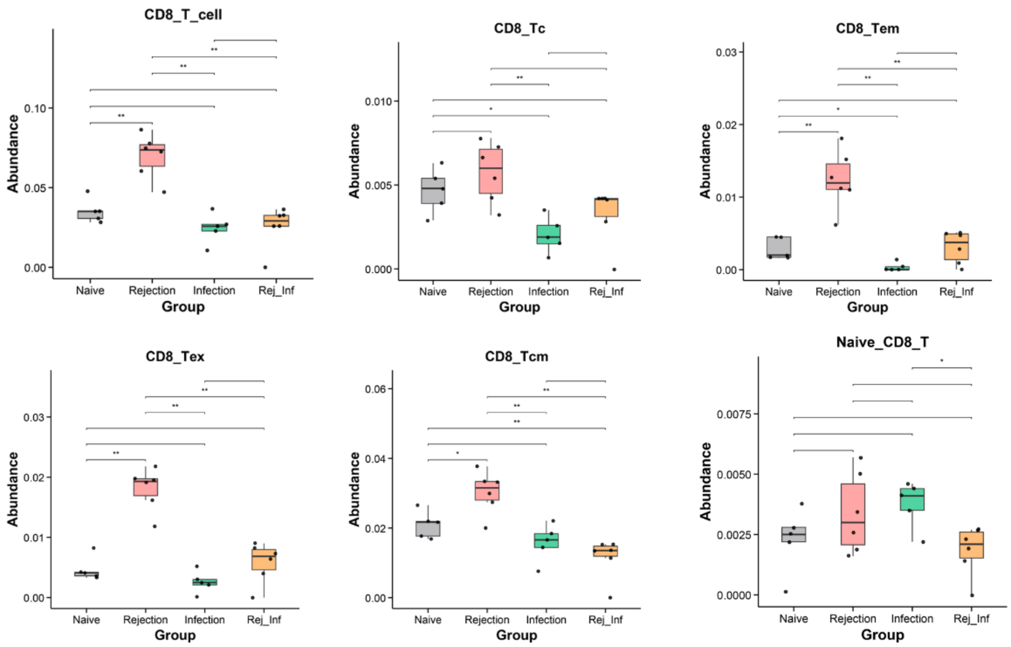

Figure S21. Boxplots show the abundance of CD8+ T cell, CD8+ cytotoxic T cell (CD8_Tc), CD8+ effector memory T cell (CD8_Tem), CD8+ exhausted T cell (CD8_Tex), CD8+ central memory T cell (CD8_Tcm), and naive CD8+ T cell in the Naive, Rejection, Infection, and Rej-Inf groups.

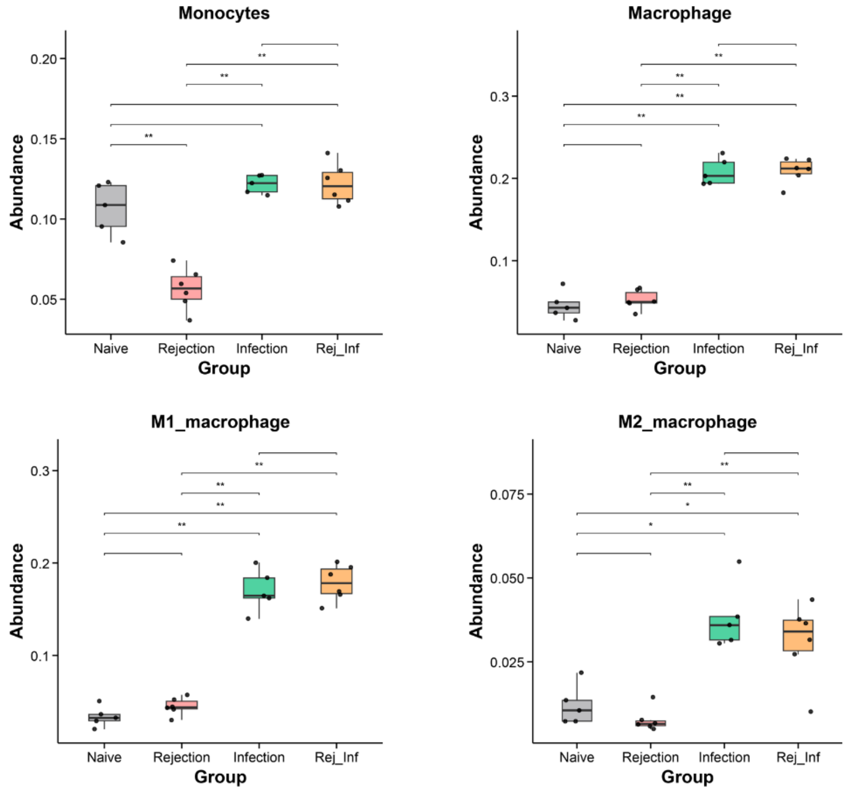

Figure S22. Boxplots show the abundance of monocytes, macrophages, M1 macrophages, and M2 macrophages in the Naive, Rejection, Infection, and Rej-Inf groups.

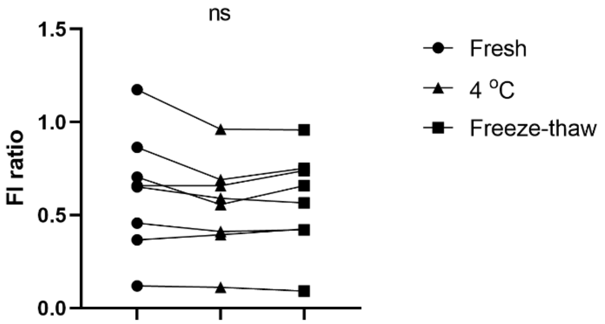

Figure S23. Pre-analytical robustness of Caliper-17 assay using clinical samples. Statistical significance was assessed using repeated-measures one-way ANOVA with Greenhouse–Geisser correction. ns: no significance.
